# Lose-lose consequences of bacterial community-driven invasions in soil

**DOI:** 10.1186/s40168-024-01763-7

**Published:** 2024-03-18

**Authors:** Xipeng Liu, Joana Falcão Salles

**Affiliations:** Microbial Ecology Cluster, Genomics Research in Ecology and Evolution in Nature (GREEN), Groningen Institute for Evolutionary Life Sciences (GELIFES), University of Groningen, 9747 AG Groningen, The Netherlands

**Keywords:** Microbial interactions, Biotic disturbances, Beneficial microbial consortia, Niche overlap and divergence, Functional redundancy, Soil restoration

## Abstract

**Background:**

Community-driven invasion, also known as community coalescence, occurs widely in natural ecosystems. Despite that, our knowledge about the process and mechanisms controlling community-driven invasion in soil ecosystems is lacking. Here, we performed a set of coalescence experiments in soil microcosms and assessed impacts up to 60 days after coalescence by quantifying multiple traits (compositional, functional, and metabolic) of the invasive and coalescent communities.

**Results:**

Our results showed that coalescences significantly triggered changes in the resident community's succession trajectory and functionality (carbohydrate metabolism), even when the size of the invasive community is small (~ 5% of the resident density) and 99% of the invaders failed to survive. The invasion impact was mainly due to the high suppression of constant residents (65% on average), leading to a lose-lose situation where both invaders and residents suffered with coalescence. Our results showed that surviving residents could benefit from the coalescence, which supports the theory of “competition-driven niche segregation” at the microbial community level. Furthermore, the result showed that both short- and long-term coalescence effects were predicted by similarity and unevenness indexes of compositional, functional, and metabolic traits of invasive communities. This indicates the power of multi-level traits in monitoring microbial community succession. In contrast, the varied importance of different levels of traits suggests that competitive processes depend on the composition of the invasive community.

**Conclusions:**

Our results shed light on the process and consequence of community coalescences and highlight that resource competition between invaders and residents plays a critical role in soil microbial community coalescences. These findings provide valuable insights for understanding and predicting soil microbial community succession in frequently disturbed natural and agroecosystems.

Video Abstract

**Graphical Abstract:**

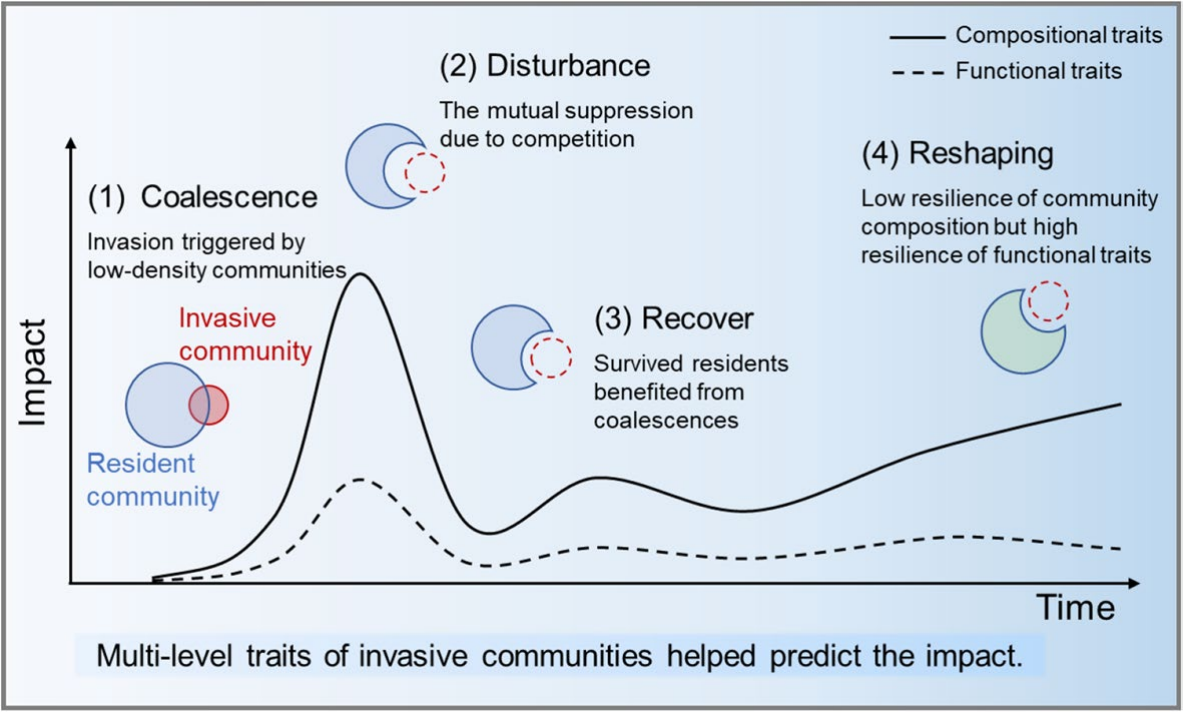

**Supplementary Information:**

The online version contains supplementary material available at 10.1186/s40168-024-01763-7.

## Background

Microbial invasion is a thriving area in microbial ecology focusing on the introduction, establishment, and impact of alien microorganisms on the resident microbial community [1]. Similar to the invasion of larger organisms, which poses a significant threat to the extinction of resident species and ecosystem functioning [2], microbial invasions regulate microbiomes’ assembly, dynamics, and stability [3–5]. The initiator of microbial invasion could be a single invader or a whole community, the latter also known as community coalescence (i.e., community-driven invasion) [6]. While mixing entire communities is hard to envisage in plant and animal ecology, microbial community coalescence is likely an ever-present feature, occurring widely in natural ecosystems [7]. However, contrary to examples from the aquatic literature [8, 9], we lack studies on soil community-driven invasion despite its ubiquity and importance in soil ecosystems.

Community coalescences frequently occur when a soil microbial community is introduced outside its geographic boundaries and merged with others due to water and wind flow, animal migration, or human activities [10, 11]. On a large geographic scale, distance-decay relationships suggest that such community-driven invasion due to microbial dispersal widely shapes microbial biogeographic patterns at shorter distances [12–14]. This evidence suggests that community coalescence in the soil can significantly change the resident community composition and functionality, at least on short spatial and temporal scales. Coalescence can also occur in soil agroecosystems, where microbial consortia are deliberately introduced into the soil to achieve more efficient bioremediation, biocontrol, and bio-fertilization [7, 15–17]. Overall, these scenarios highlight the importance of community-driven invasion in terrestrial ecosystems and beg two fundamental questions: whether and how invasive species survive in their recipient soil and how introduced microbiomes affect the resident microbial community. Furthermore, considering the increasing use of microbial inoculants and consortia [18–20], it is crucial to understand how biotic disturbances influence soil microbial interactions and their potential impact on soil ecosystem functions and services.

Our current knowledge of community-driven invasions and their legacy derives from aquatic environments, in silico and in vitro approaches, or single-invader soil studies. It is widely accepted that soil microbial communities are not resilient to invasion by a single invader, both in the context of the inoculation of beneficial microorganisms in agricultural settings and non-soilborne invaders (e.g., *Escherichia coli*) [21–25]. Generally, the invader’s survival in soil follows a progressive decline due to the selection pressure imposed by both resident taxa and the environment. Despite this decline, single invaders leave a footprint on the native resident community (in terms of diversity reduction and compositional shift), and this impact is positively related to their survival rate [23, 25]. The effect of a single invader on the structure and function of native communities is likely long-lasting even if the invader becomes extinct [24, 26]. In the scenario of community-driven invasion, however, different invaders from an invasive community may show various survival patterns [27, 28]. Thus, if community coalescence follows the evidence demonstrated for single invaders, the impact of community coalescence on the resident community might be remarkable and challenging to predict.

Regarding the mechanism underlying microbial invasions, niche (resources) competition between an invasive species and resident taxa has been demonstrated to play a significant role, where intense competition suppresses the invader’s establishment and causes impacts on the resident community [22, 29, 30]. For example, the exogenous addition of carbon source used by a single invader (e.g., *E. coli*) could increase its survival in the soil [22]. In addition, a recent in silico research suggested that reducing competition between parent communities led to coalescent communities with higher species richness [31]. However, the extent to which this mechanism applies to community-driven invasions in the soil is unclear, where interspecies interactions within and between communities may amplify the inherent complexity of community niche or resource utilization characteristics, and further influence the process and outcome of the community coalescence.

This study explores the process and mechanism of microbial community-driven invasion in the soil. We performed a soil microcosm experiment where nine invasive soil communities sharing the same bacterial density but differing in diversity (species richness) and composition were introduced separately as invasive communities into different microcosms containing the same natural soil. Using amplicon and shotgun metagenomic sequencing and Biolog MicroPlates, we assessed invasion impact up to 60 days after coalescence (invasion) and linked them to multiple traits (compositional, functional, and metabolic) of invasive and resident communities. Following observations of single invaders, we hypothesized that the succession trajectory of soil microbial communities would vary depending on the alien communities and that invasion consequences would be mainly regulated by the competition between invasive and resident taxa for the available niches.

## Methods

### Construction of invasive communities and the resident community

**Fig. 1:**
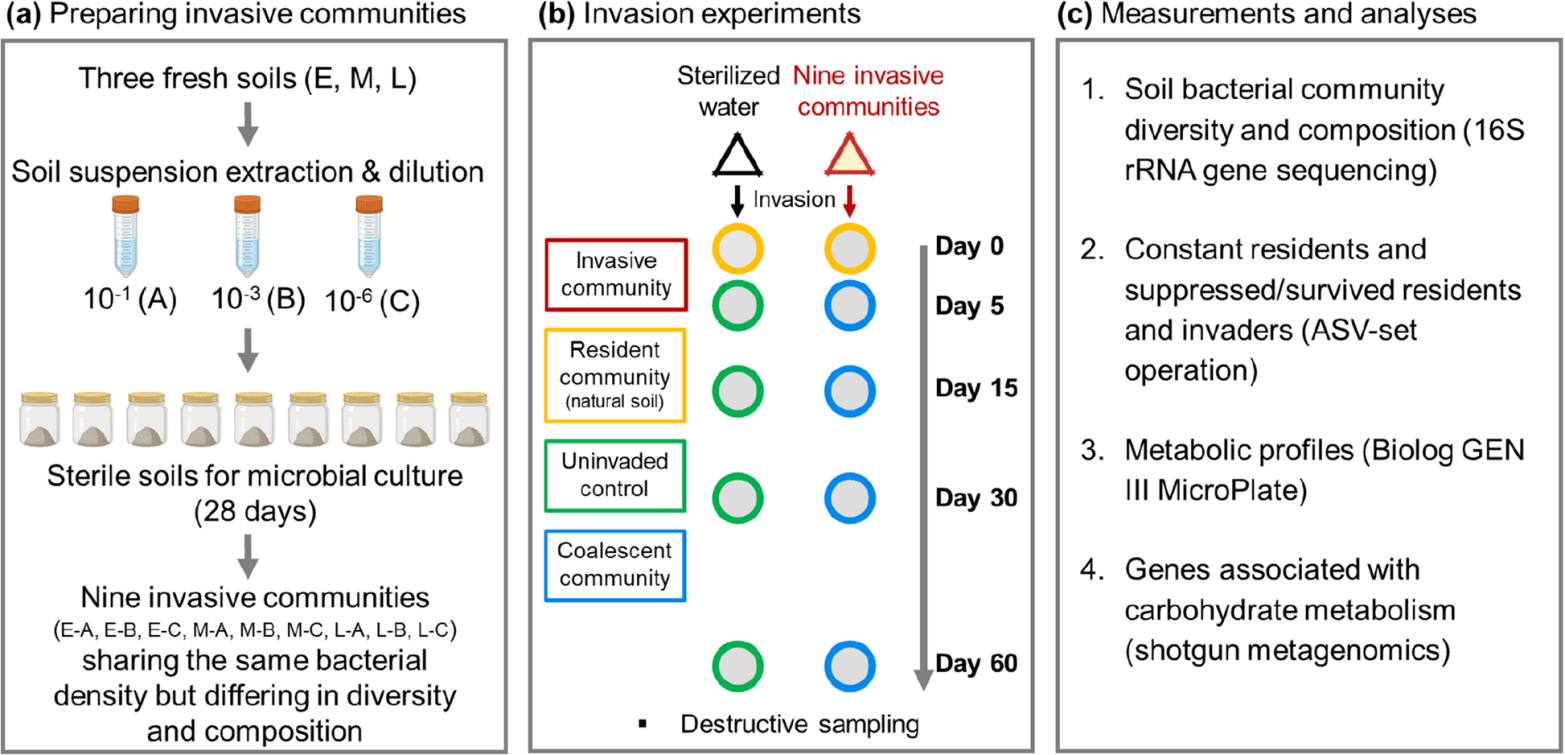
Schematic illustration of the experimental design. **a** We created nine invasive communities differing in composition and diversity (species richness) by inoculating diluted soil suspensions (A: 10^−1^, B: 10^−3^, and C: 10^−6^) extracted from three soils (E, M, and L, collected from salt marsh locations showing a significant difference in composition) in sterile soils obtained from an agricultural field. These were incubated for 28 days to allow for soil colonization and similar cell densities. At the end of this period, we generated nine soil communities that differed in species richness and composition but had similar cell densities. **b** Invasion experiments were performed by introducing nine invasive communities that had been adjusted to the same bacterial density into the soil collected from late-stage salt marsh and containing a natural, original resident community, soil L). The experiment was designed to consist of five destructive samplings on days 0, 5, 15, 30, and 60 after the invasion, using three replicates for each invasion treatment at each date. **c** Summary of the main analyses and respective measurements performed in this study

A total of nine invasive communities were constructed in this experiment (Fig. 1). For that, we initially collected soils from three different locations along a primary succession (E, early; M, middle; and L, late succession) located on a salt marsh ecosystem at the island of Schiermonnikoog, the Netherlands (53°30′ N, 6°10′ E). The microbial communities of these soils were well described in earlier studies and showed significant differences in composition [32, 33]. Fresh soils used in this study were collected on 21 June 2021 and were stored at 4 °C after sieving through the 2-mm sieve. The subsequent preparations associated with the invasive and resident communities started on 26 June 2021.

To obtain invasive communities, the 20 g of soils (E, M, and L) were resuspended in 180 ml sterilized water and further diluted to the 10^–6^ factor by a tenfold dilution procedure. The procedure was applied to induce changes in the species richness of the diluted communities by serially removing rare species [24, 30]. Next, 50 ml of the soil suspensions from the 10^−1^, 10^−3^, and 10^−6^ dilutions were inoculated into 120 g of sterile soil, respectively, generating nine microcosms that were incubated for 28 days at 26 °C to allow for microbial colonization. The sterile soil was prepared by collecting fresh soil from a potato field (sandy loam, pH 4.75) in Friesland, the Netherlands, and gamma irradiating (50 kGy) it [34]. After the 28-day incubation, the soil microbiome in these nine microcosms, which differed in diversity and composition but with similar cell density, were transferred to 0.85% saline solution, generating 1:10 soil suspensions that were used as invasive communities. Therefore, we refer to the dilution gradient 10^−1^, 10^−3^, and 10^−6^ as A, B, and C. Each coalescence treatment is written as E-A, E-B, E-C, M-A, M-B, M-C, L-A, L-B, and L-C, with the first letter representing the different soil origin and the second one the dilution levels.

Soil microcosms containing the resident community were constructed in sterile and covered glass jars (6 cm in diameter, 10 cm in height) containing 50 g of sieved natural soil, collected from the late-stage succession (Soil L) in Schiermonnikoog [32, 33]. Soil water content in all microcosms was adjusted to 75% water-holding capacity. Before the coalescence experiments, the soil was pre-incubated for 30 days at 26 °C to stabilize the microbial community.

### Set-up and design of coalescence experiments

The coalescence experiment consisted of introducing the soil suspensions (extracted with 0.85% saline solution) obtained from the nine invasive communities into resident communities (Fig. 1c). Notably, the total bacterial density of all invasive communities was adjusted to 5% of that in the resident community, following densities used in previous studies focusing on single-invader invasions [22, 24]. To achieve this, the bacterial density of all invasive and resident communities used in this experiment was measured in advance by checking total culturable bacteria on R2A agar for 3 days (see Supplementary Table S1 for details on soil bacterial density of invasive and resident communities) and adjusted to same density in soil suspensions with sterile water. We, therefore, introduced nine invasive communities containing the same density of bacterial cells but differing in diversity and composition into native, resident communities (Supplementary Fig. S1). The uninvaded control was set by adding sterile water with the same volume as the other nine treatments. The experiment was designed for five destructive samplings on days 0, 5, 15, 30, and 60 after the coalescence, using three replicates for each coalescence treatment at each date. Thus, we constructed 150 soil microcosms: ten treatments (nine invasive communities and one uninvaded control) × three replicates × five sampling dates. For convenience, we named the resident community on day 0 as the original community (before coalescence) and the uninvaded resident communities on days 5, 15, 30, and 60 as the uninvaded control. The resident communities subjected to invasion by treatments E-A, E-B, E-C, M-A, M-B, M-C, L-A, L-B, and L-C are called coalescent communities, following the treatment identifiers.

After coalescence, all microcosms were incubated under a constant temperature (26 °C) in darkness. Each microcosm was covered with aluminum foil to prevent contaminants but allowed ventilation. During the experiment, the soil moisture in all microcosms was kept at 75% water-holding capacity.

### Amplicon sequencing and bioinformatic processing

The microbial genomic DNA was extracted from 153 soil samples (the extra three samples were taken from fresh soil L before the lab incubation). In brief, 0.25 g of soil was used to isolate DNA with the DNeasy PowerSoil Pro Kit (QIAGEN, Germany) according to the manufacturer’s instructions. DNA concentration was quantified using a NanoDrop 2000 Spectrophotometer (Thermo Fisher Scientific, Waltham, MA, USA). The prokaryotic genomic libraries were prepared and sequenced at the University of Minnesota Genomics Center (UMGC) following the two-step dual-indexing approach [35]. The produced 16S rRNA gene_V4 amplicons constructed with primer pair 515F (5′-GTGCCAGCMGCCGCGGTAA-3′), and 806R (5′-GGACTACHVGGGTWTCTAAT-3′) were sequenced by 2 × 300 bp MiSeq at UMGC to characterize prokaryotic communities.

We used the QIIME2 pipeline (version 2020.8) to process 16S rRNA gene sequencing data. First, the quality control and removal of low-quality regions of the sequences were performed with DADA2 to infer Amplicon Sequence Variants (ASVs). Next, taxonomy was assigned to representative sequences using the Silva 138 Naive Bayes 515F/806R classifier. All ASVs affiliated with archaea, eukaryotes, mitochondria, and chloroplast were removed from the dataset. Finally, 4,180,791 sequences were obtained from all 153 soil samples. The pipeline FastTree generated a phylogenetic tree from representative sequences by aligning sequence fragments via the MAFFT program. To make samples comparable, the feature table of each sample was rarefied to a depth of 8178 sequences. Six samples containing a relatively small number of sequences were thus filtered.

### Shotgun metagenomic sequencing and bioinformatic processing

We selected 54 genomic DNA samples (treatments E-A, M-A, L-A, L-B, L-C, and control on days 0, 5, and 60 with three replicates) for shotgun metagenomic sequencing. To assess the functional attributes of microbial communities, total genomic DNA samples were sent to BGI TECH SOLUTIONS (HONGKONG) CO., LIMITED, Hong Kong, to construct the library and perform the shotgun sequencing. The Short-Insert library was used and sequenced on a 2 × 150-bp DNBseq platform.

Raw reads were preprocessed by removing adaptor sequences, contamination, and low-quality reads with SOAPnuke [36] to obtain clean reads (39,615,462 reads per sample on average). Megahit v. 1.2.9 was used to perform de novo assembly for each sample with the k-mer length increasing from 21 to 149 in steps of 20 [37]. Assembled contigs over 500 bp were submitted to Prodigal v. 2.6 to predict protein-coding genes [38]. After discarding genes shorter than 100 bp, genes from all 54 samples were clustered at ≥ 95% identity and ≥ 90% overlap with MMseqs2 [39], resulting in a catalog containing 36,889,801 non-redundant genes. The translated proteins of all non-redundant genes were annotated by searching against enzymes involved in carbohydrate metabolism using the carbohydrate-active enzymes (CAZy) database via DIAMOND BLASTp (options: -k 1 -e 1E-5) [40]. Paired-end reads of each sample were mapped to the gene catalog using Salmon v.1.9.0 [41]. The relative abundance (estimated as Transcripts Per Million, TPM, by Salmon [41]) of each gene encoding for carbohydrate-active enzymes was summed by gene subfamily and used in downstream analyses.

### Distinguishing the survived and suppressed invaders and residents

To obtain direct insight into the invasion impacts on the resident community, we performed ASV-set operations to distinguish the survived and suppressed invaders and residents (Supplementary Fig. S2). First, the *constant resident* was defined as the resident taxa that remained in the soil during incubations without invasions. The constant resident could thus be detected by intersecting the original community L with the uninvaded control at each sampling date (Label 1 in Supplementary Fig. S2a). The *survived resident* (Label 5 in Supplementary Fig. S2a) represents the native taxa present in the soil after the coalescence. It can be detected by the intersection of constant residents and coalescent communities in the invasive community. Similarly, the *suppressed resident* (Label 1 in Supplementary Fig. S2a) corresponds to the native taxa that are below the detection limit after coalescence.

The *survived invaders* (Label 6 in Supplementary Fig. S2a) represent invasive taxa that successfully colonized the soils upon coalescence. In contrast, the *suppressed invaders* (Label 2 in Supplementary Fig. S2a) refer to invasive taxa that were present in the invasive communities but were found below the detection limit or did not survive coalescence. Note that the other ASVs detected in invasive and coalescent/constant communities (i.e., Labels 3 and 4 in Supplementary Fig. S2a) were not considered due to the vague definition. Moreover, these ASVs only comprise a tiny percentage (< 0.5%) of invasive communities. The detailed calculation method is shown in Supplementary Fig. S2b. Replicates of each treatment was combined into one ASV set for these analyses. Based on the result obtained above, we calculated the suppression rate of the resident community, representing how many residents were suppressed by coalescence, and the invaders' survival rate, indicating how many species from the invasive communities survived.

### Quantifying metabolic traits of microbial communities

Metabolic traits (i.e., the pattern and amount of carbon sources used) of soil microbiome were assessed by measuring the potential metabolic activity for an array of 71 different (organic) carbon (C) sources using the Biolog GEN III MicroPlate (Biolog, Hayward, California, USA). The 71 carbon sources were grouped into carbohydrates, carboxylic and acetic acids, and amino acids. The assessment protocol was described in previous studies and was in accordance with Biolog’s high cell density phenotypic microarray protocol [24, 42]. The substrate utilization was calculated using the area under the utilization curve, and the maximum value for the plate was used to normalize the data across all 71 C sources. These data were used to calculate the metabolic unevenness index (uneven utilization of 71 C sources) and metabolic similarity (pairwise comparison of the consumed C sources and their abundances across samples, using Bray-Curtis dissimilarity). These measurements and analyses were performed on day 0 (before the invasion, for invasive communities and the original/native communities) and day 30 (after the invasion, for coalescent communities and the uninvaded control).

### Statistical analyses

Analyses and visualizations were mainly performed in R (Version 4.2.2). Spearman correlation analyses were used in this study for the linear regression fitting, and the significance was tested with the “ggpubr” package [43]. Correlation matrix was visualized using Origin (Version 2018). Two-way ANOVA analyses with invasive community treatment and time as factors were used to examine differences in the diversity of coalescent communities. One-way ANOVA with Tukey's HSD was used to assess the differences between treatments (for richness and unevenness, etc.). The differences in community composition were tested using Permutational Multivariate Analysis of Variance (Adonis) with package “vegan” [44]. The means of the two groups were compared with the “ggpubr” package [43], combining the one-way ANOVA and (paired) *t*-test. Strong’s dominance index was used to represent the unevenness index of microbial community traits and was calculated using the “abdiv” package [45]. Dissimilarity/similarity values based on Bray-Curtis were calculated using the R package “vegan” [44]. The phylogenetic distance between sub-communities (survived/suppressed residents) and their corresponding invasive communities was calculated using the R package “phyloseq” [46]. The statistically significant carbon sources driving the community changes in richness and composition were assessed in Random Forest analysis (*n* = 27) with the “*rfPermute*” package [47]. Finally, the fold change of each carbohydrate-active gene was calculated and statistically tested by the t-test. Error bars in all figures of this study indicated the standard deviation.

## Results

### Diversity and composition of invasive and resident communities

Nine invasive communities were created by introducing and inoculating different soil suspensions in sterile soil for 28 days. Compared with the resident community, all invasive communities had significantly lower diversity but higher culturable bacterial density (*p* < 0.05, Tukey’s HSD; Supplementary Table S1 and Fig. S1a and b). The composition between resident and invasive communities differed significantly (*p* < 0.001, Adonis; Supplementary Fig. S1c and d). For example, Pseudomonadota, Actinobacteriota, Chloroflexota, and Planctomycetota shared similar relative abundance and dominated in the resident community, whereas Pseudomonadota and Bacillota were the most abundant phyla in invasive communities (Supplementary Fig. S1d).

**Fig. 2:**
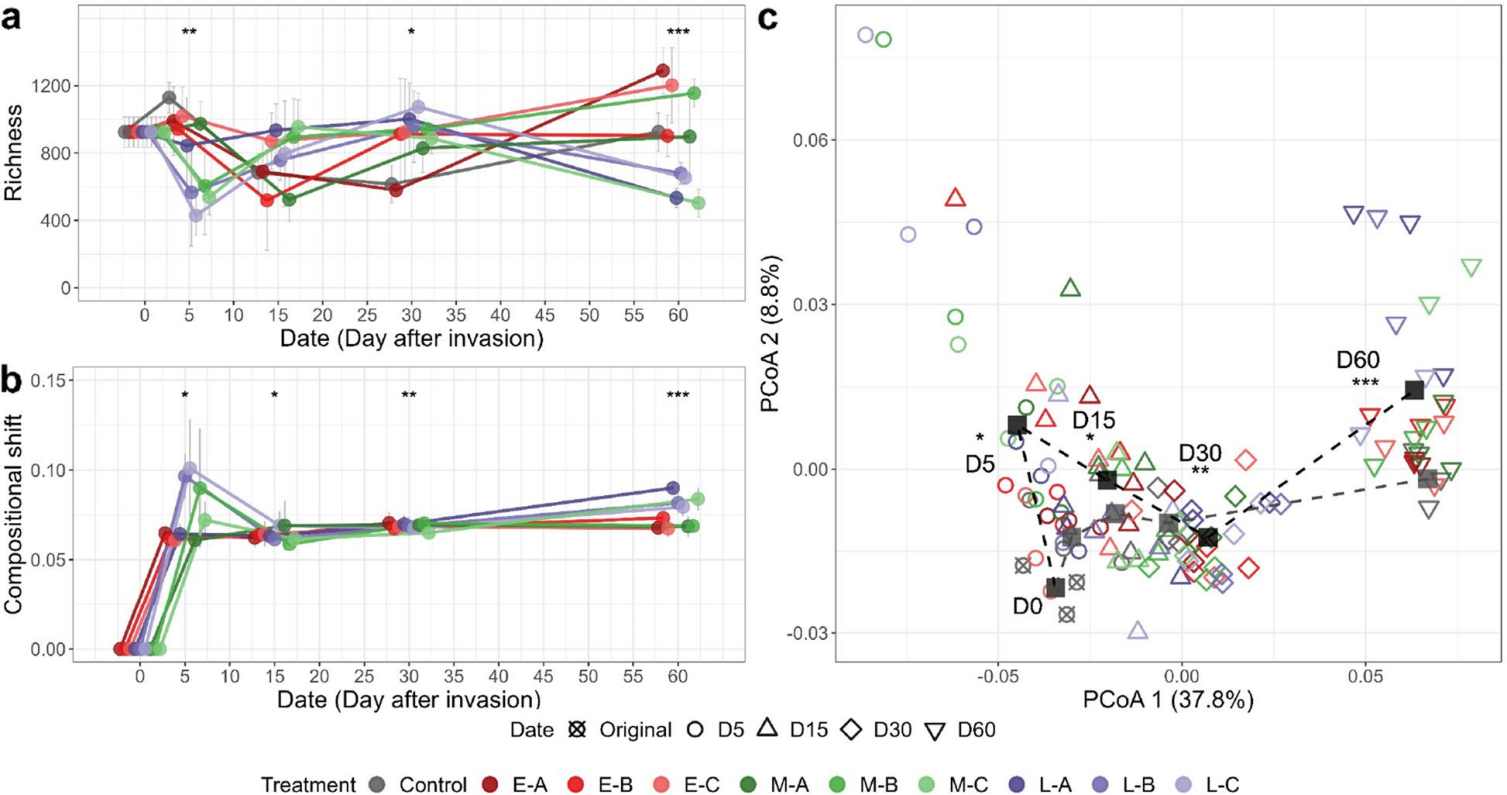
Community-driven invasions trigger changes in the successional trajectories of soil bacterial communities. **a** ASV richness of treatments across 60 days. **b** The compositional shift of coalescent communities compared to the uninvaded control based on weighted UniFrac distance. Points in figures were dodged horizontally to prevent overlapping. **c** Principal Coordinates Analysis (PCoA) shows the succession of community composition between coalescent and uninvaded communities across time. Black and gray squares represent the average status of coalescent and uninvaded treatments, respectively. Asterisks (“*”, *p* < 0.05; “**”, *p* < 0.01; “***”, *p* < 0.001) indicate significant differences among treatments for richness (one-way ANOVA with Tukey’s HSD) and composition (Adonis test)

The diversity and composition of coalescent communities fluctuated dramatically after invasions, and such changes depended on invasive communities (Fig. 2). Our results showed that the richness of coalescent communities significantly changed over time, invasive community treatment, and was influenced by the interaction between treatment and time (two-way ANOVA,* p* < 0.001) (Supplementary Table S2). Zooming in on each date, the richness of coalescent communities showed significant variance among treatments on day 5, 30, and 60 after coalescences (one-way ANOVA,* p* < 0.05; Fig. 2a). For instance, on day 5 after coalescence, ASV-richness in all treatments decreased compared to the control, in which statistical significance was observed in M-B (~ 46%), M-C (~ 52%), and L-C (~ 62%) treatments (Tukey’s HSD,* p* < 0.05). On day 60, after the coalescence, the average diversity of all treatments returned to a level comparable to the control but showed a clear divergence among treatments (Fig. 2a). Regarding the community composition, significant differences among treatments were observed for all four dates (Adonis, *p* < 0.05; Fig. 2b) in which the larger variance occurred on days 30 and 60.

Coalescence by different invasive communities induced successional trajectories in the resident communities, and this effect was more significant over time (Fig. 2c), as indicated by the increase in the successional path length, defined as weighted UniFrac distance between two adjacent time points of coalescent communities during the first 30 days (D0-D30) compared with the uninvaded control (Supplementary Fig. S3).

### Coalescence leads to high suppression of invasive communities

The survival rate of the invasive community was lower than 1% in all treatments, which means that most invaders did not survive (or were below the detection limit) post-coalescence (Supplementary Fig. 4a). Moreover, there were no significant differences in survival rates and abundance among the four dates (Supplementary Fig. S4c). The average relative abundance of survived invaders was approximately 0.1% of the coalescent community. Most survived invaders were affiliated with Bacillota (accounting for 65–91% among four dates), in which *Bacillus* accounted for 40% (Supplementary Fig. S4d). A Venn diagram showed that these survived invaders were detected at different sampling dates (Supplementary Fig. S4e), indicating that their survival was not due to a stochastic process. The relationship between invaders’ abundance in the invasive and coalescent communities was analyzed to investigate whether a higher initial abundance of invaders can boost their survival (Supplementary Fig. S5). The significant and positive Poisson regression was observed on days 5 and 30 (*p* = 0.014 and 0.0051, respectively) but failed to fit with this model on day 15 (*p* = 0.75). On day 60, we found a negative correlation of invaders’ abundance (*p* = 0.0067) in the invasive and coalescent communities (Supplementary Fig. S5).

### Suppression of residents contributes to invasion impacts

**Fig. 3:**
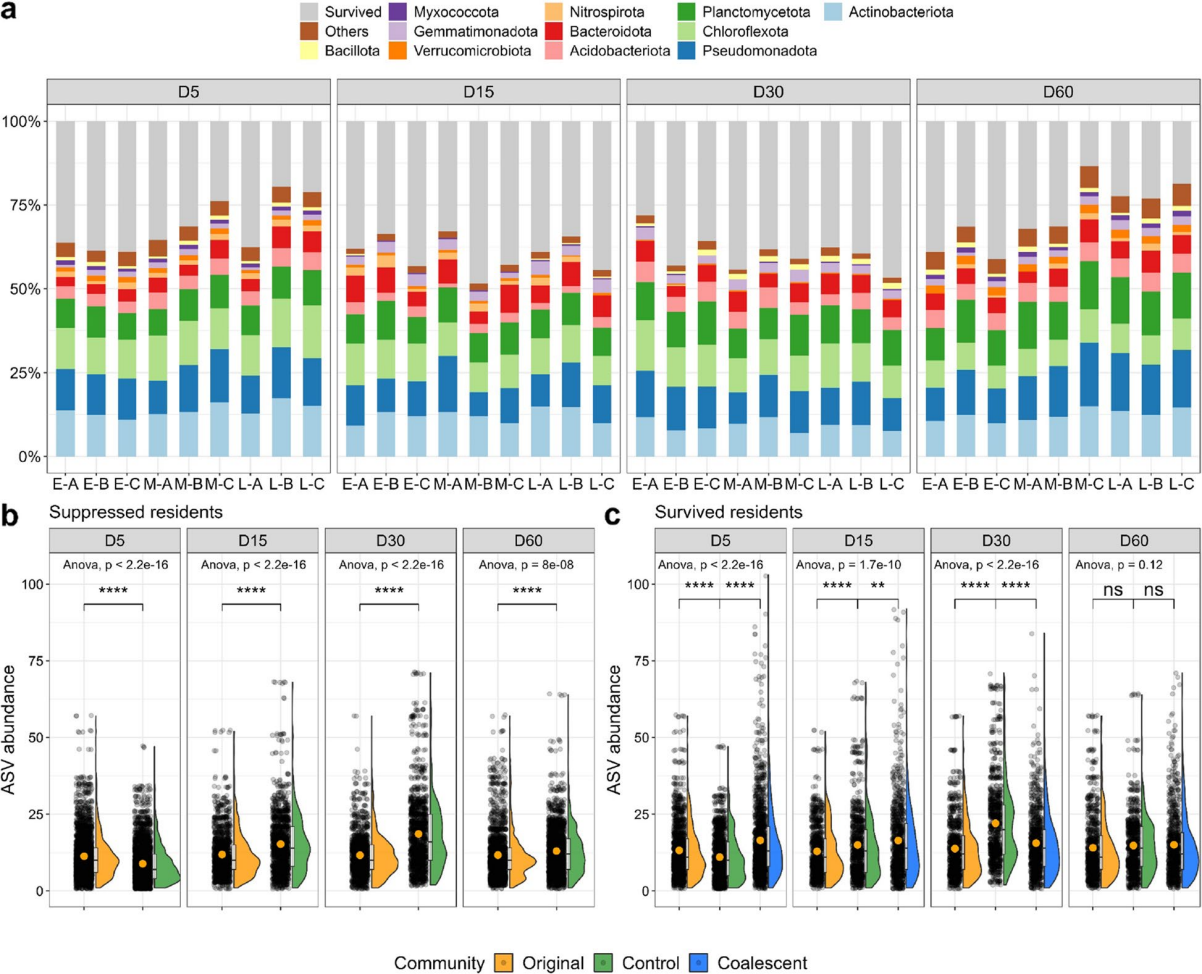
The survival and suppression of resident taxa after coalescence. **a** The proportion of suppressed/survived taxa in the resident sub-community. The gray color indicates the taxa that survived coalescence, while other colors represent suppressed ones. **b** Represents the abundance of ASVs (number of reads) present in the original communities and the uninvaded control that were suppressed by coalescence at each sampling time. **c** Represents the abundance of ASVs (number of reads) present in the original communities and uninvaded controls that survived coalescence at each sampling time. Each dot corresponds to the abundance of a specific ASV in each community. The orange dots indicate the mean value. On average, the number of resident ASVs that survived coalescence increased upon invasion, at the first 15 days of coalescence, as they were present in lower numbers in the original and uninvaded control. One-way ANOVA and *p* value indicate the global variance among groups. Asterisks above the violin plot represent the significant difference in the mean abundance of ASVs between different communities (“ns”, *p* > 0.05, “*”, *p* < 0.05, “**”, *p* < 0.01, “***”, *p* < 0.001, “****”, *p* < 0.0001; paired *t*-test)

To disentangle the invasion-driven changes in coalescent communities, we used ASV-set operation to identify the number and identity of the residents that survived or were suppressed in each treatment and on each date after the invasion when compared to the uninvaded control (Fig. 3). For the native residents, an average of 65% of the taxa was suppressed to below the detection limit for all four dates upon invasion (Fig. 3a). On day 5, the resident communities that were coalescent by L-B, L-C, and M-C showed the highest suppression rate (> 75%) (Fig. 3a). The suppression rate under these three treatments then decreased on days 15 and 30 with the increasing survival rate of residents, eventually achieving a higher suppression value (> 75%) again on day 60. From a taxonomy perspective, the most suppressed resident taxa belonged to the phyla Actinobacteriota, Pseudomonadota, Chloroflexota, and Planctomycetota, which aligned with the dominant phyla in the original community (Figs. 3a and S1d). We observed that the suppressed residents had a higher average abundance in the uninvaded control on days 15, 30, and 60 than that in the original community (day 0) (Fig. 3b). This indicates that coalescence led to a decrease in the abundance of ASVs that would otherwise have remained more abundant in the absence of invasion (uninvaded control). Furthermore, we observed that the surviving residents showed a significantly higher mean abundance in coalescent communities than in the uninvaded control on days 5 and 15 (Fig. 3c), indicating that the survived taxa benefited from coalescences.

**Fig. 4:**
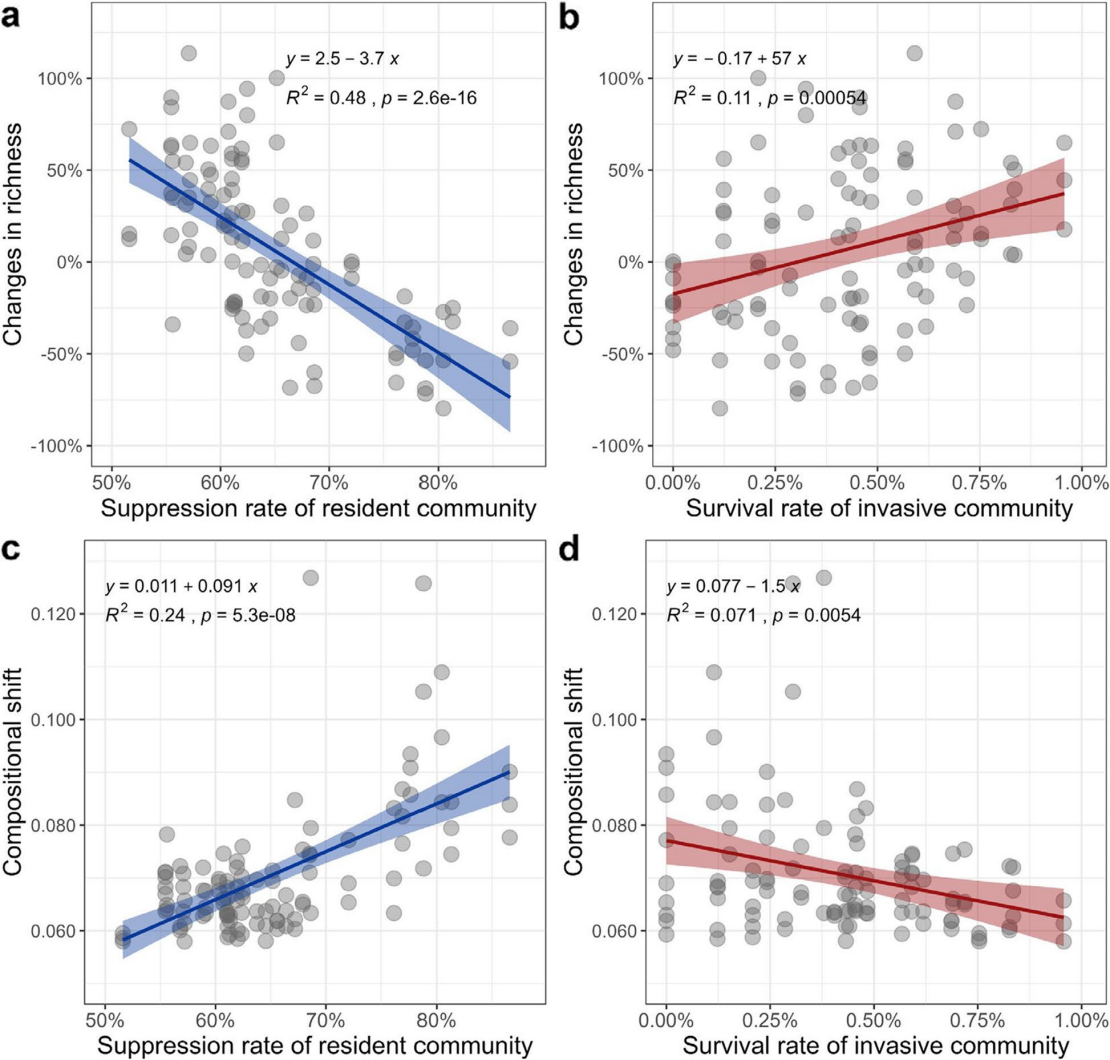
Contributions of the survival and suppression of residents and invaders to invasion impacts. Regression analyses showing the relationship between changes in diversity (between coalescent communities and uninvaded controls) and the suppression rate of the resident community (**a**), and the survival rate of the invasive community (number survived invaders / total number species in invasive communities) (**b**). Regression analyses showing the relationship between the compositional shift between coalescent communities and uninvaded controls and the suppression rate of the resident community (**c**), and the survival rate of the invasive community (**d**) within 60 days. The percentage of changes in richness and the dissimilarities (based on weighted UniFrac distance) in compositional shifts of the coalescent communities were calculated in relation to the uninvaded control for each sampling time. Changes in diversity between coalescent communities and uninvaded controls were calculated according to the formula: [(richness coalescent community - richness uninvaded control)/richness uninvaded control]). The suppression rate of the resident community refers to the number of suppressed residents divided by the total number of residents in uninvaded control. The survival rate of the invasive community refers to the number of survived invaders divided by the total number of species in invasive communities

The observed changes in the α-diversity of coalescent communities compared to uninvaded control [(richness coalescent community - richness uninvaded control)/richness uninvaded control] were negatively and significantly correlated with the suppression rate of resident communities across four sampling dates (Fig. 4a; *R*^2^ = 0.48, *p* < 0.00001). In contrast, there was a positive and significant correlation between richness changes and the survival rate of the invasive community, although with a relatively lower correlation coefficient (Fig. 4b, *R*^2^ = 0.11, *p* < 0.001). These results indicate that the change in richness is mainly due to the suppression of the resident communities rather than the survival of invaders.

Furthermore, the suppression rate of the resident community was also significantly correlated with the compositional dissimilarity between the coalescent community and the uninvaded control (Fig. 4c, *R*^2^ = 0.24, *p* < 0.00001). However, the survival rate of the invasive community has a significant and negative correlation with the compositional shift of the coalescent community upon invasions (Fig. 4d, *R*^2^ = 0.071, *p* = 0.0073). The results of multiple linear regression analysis confirmed that the suppression rate of the resident community predicts the diversity (*R*^2^ = 0.43, *p* < 0.0001) and composition shifts (*R*^2^ = 0.36, *p* < 0.0001) in the coalesced communities better than the survival rate of the invasive community.

### Survival strategies of resident taxa

**Fig. 5:**
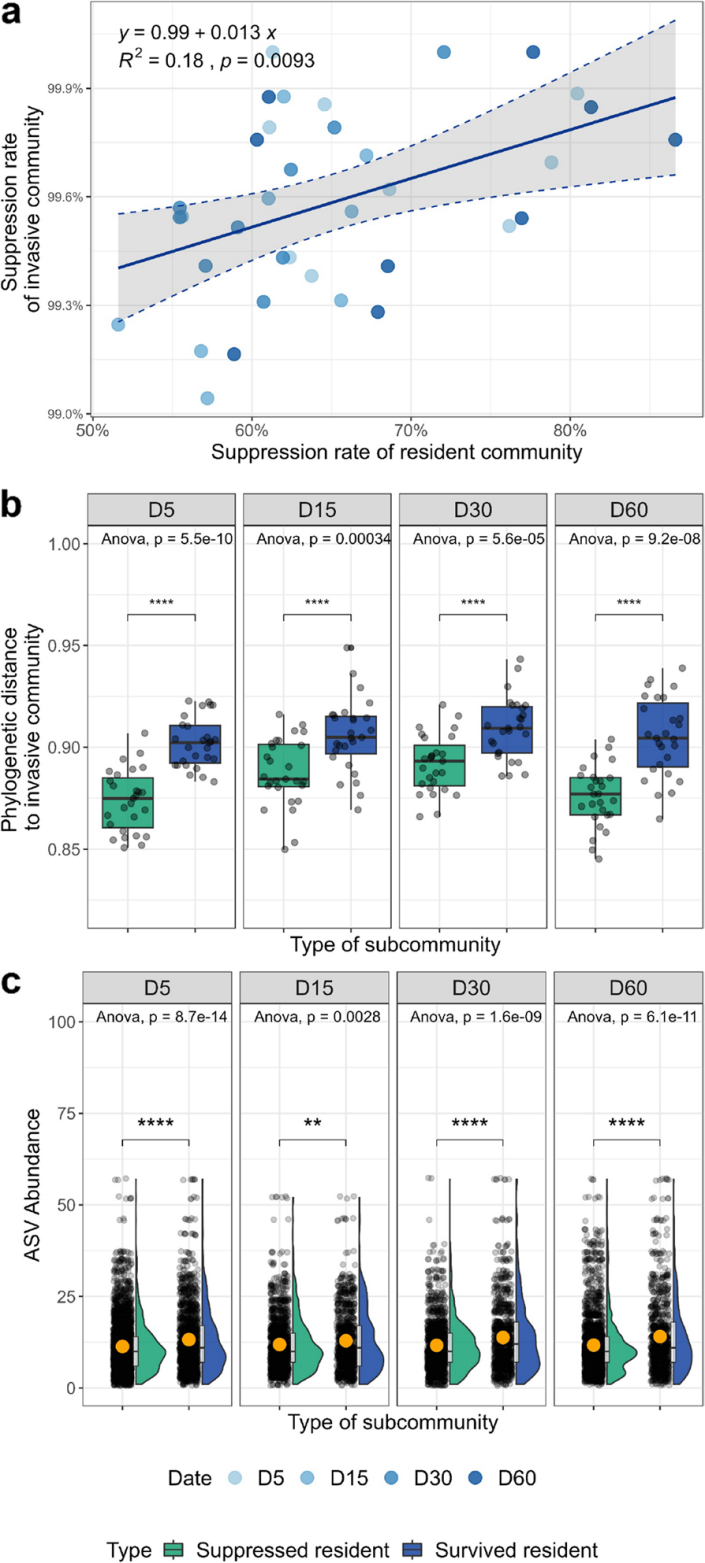
Survival strategies of resident taxa. **a** Relationship between the suppression rate of residents and invaders after coalescences. The suppression rate of invaders is the opposite of the survival rate of invasive communities. The suppression rate of the resident community and the survival rate of the invasive community were described in Fig. 3. **b** Phylogenetic distance between the invasive community and the suppressed or survived components (sub-community) of the resident communities after community coalescences. **c** The abundance (number of reads of each ASV) of the suppressed or survived resident subcommunities. An orange dot indicates the mean value. One-way ANOVA and *p* value indicate the global variance among groups. Asterisks above the dots indicate the significant difference (“ns”, *p* > 0.05, “*”, *p* < 0.05, “**”, *p* < 0.01, “***”, *p* < 0.001, “****”, *p* < 0.0001; *t*-test)

Regression analysis determined the relationship between invader and resident suppression rates for each treatment and date. The suppression rate of invaders (the opposite of invader survival) significantly increased as the suppression rate of the resident community increased (*p* = 0.0093, Spearman correlation; Fig. 5a). In other words, the mutual suppression between invaders and residents occurred after invasions. The phylogenetic distance between two sub-communities (i.e., suppressed and survived residents) and the corresponding invasive community revealed a potential dominant mechanism of species suppression upon invasions. Interestingly, we found that the phylogenetic distance between the invasive community and the suppressed residents was significantly closer than that of survived residents (*p* < 0.0001, paired *t*-test; Fig. 5b). This phenomenon, present across all four sampling dates after the invasion, suggests that phylogenetically close related species might be competing for similar resources. Besides, we observed that the mean abundance of survived resident taxa was significantly higher than that of suppressed residents for each date (*p* < 0.01, *t*-test; Fig. 5c).

### Coalescence effects on microbial functional traits

**Fig. 6:**
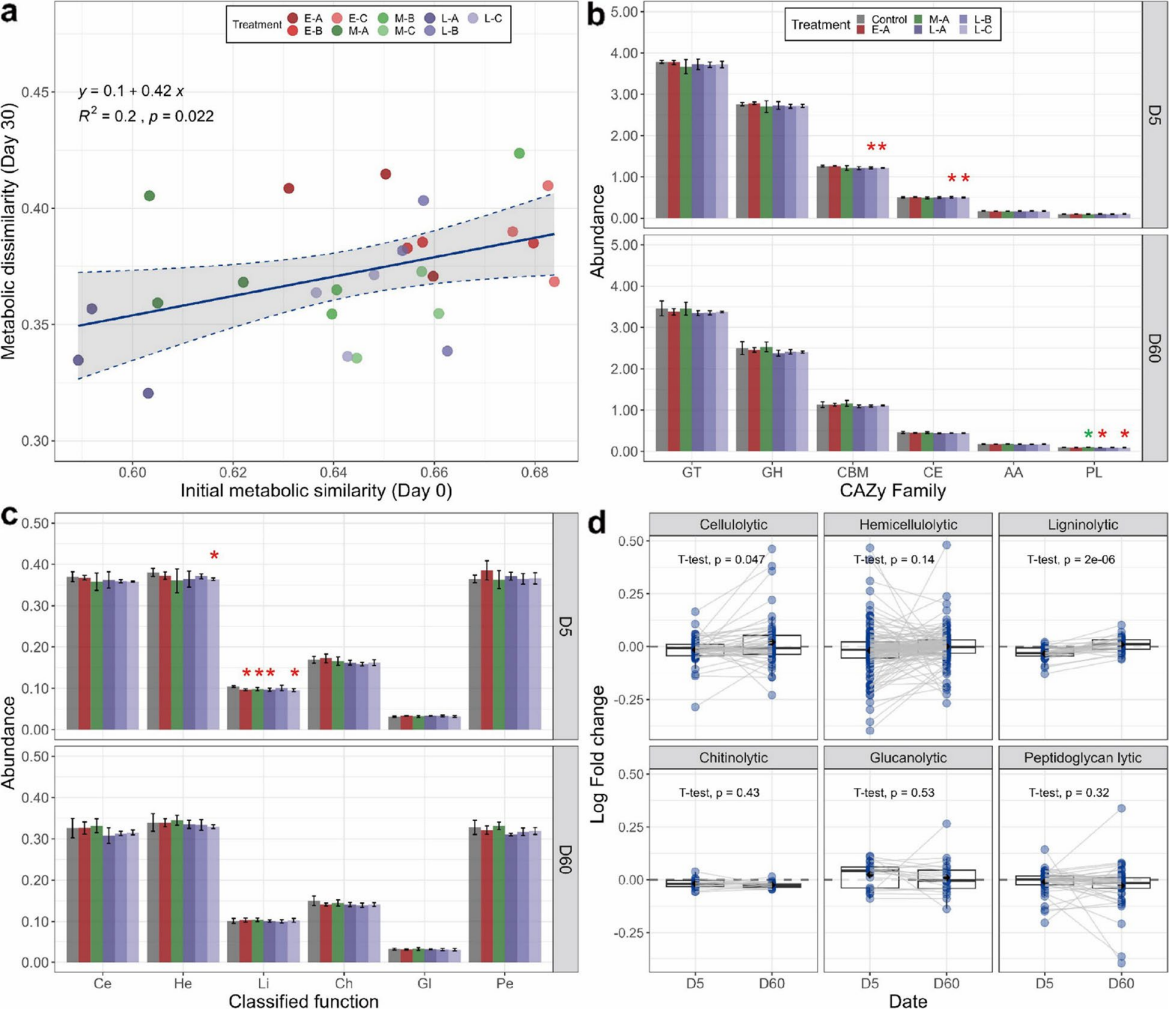
Coalescence effects on microbial functional traits. **a** Coalescence impact on metabolic profiles, assessed by Spearman’s correlation between the initial metabolic similarity (between invasive and original resident communities) and the metabolic dissimilarity between coalescent and uninvaded communities after coalescence. The (dis) similarity based on the Bray-Curtis index was calculated using the metabolism profile of 71 carbon resources for metabolic traits. **b** The abundance (TPM/1000) of CAZy gene families. GT, glycosyltransferase; GH, glycoside hydrolase; CBM, carbohydrate-binding module; CE, carbohydrate esterase; AA, auxiliary activities; PL, polysaccharide lyase. **c** The abundance of classified functions of CAZy genes. Ce, cellulolytic; He, hemicellulolytic; Li, ligninolytic; Ch, chitinolytic; Gl, glucanolytic; Pe, peptidoglycan lytic. The red and green asterisks above bars indicate a significant decreased and increase compared to the uninvaded control, respectively (“*”, *p* < 0.05, *t*-test). **d** The response (log fold change in relation to the uninvaded control) of different functions between day 5 (D5) and day 60 (D60) to invasions (paired *t*-test), suggesting the associated functioning (cellulolytic, hemicellulolytic, and ligninolytic) recovered after 60 days

After coalescence, we evaluated the impact on microbial functional traits by assessing the metabolic profile (Biolog) and functional capabilities (CAZy genes) of soil microbial communities. The metabolic unevenness was significantly higher in the resident community compared to invasive communities except for E-C (*p* < 0.05, Tukey’s HSD; Supplementary Fig. S6a). Importantly, we found that the initial metabolic similarity between invasive and resident communities was positively and significantly correlated with the functional impact of coalescence (measured by the metabolic dissimilarity between coalescent and uninvaded communities) on day 30 (*p* = 0.022, Spearman correlation; Fig. 6a). In other words, the metabolic traits of coalescent communities depend on the niche overlap between resident and invasive communities. Hence, a similar metabolic profile could cause more substantial metabolic shifts in the coalescent community.

Our results showed that the effects of invasions on the abundance of genes encoding for carbohydrate-active enzymes (CAZy) differ by enzyme families, time, and invasive communities (Supplementary Figs. S7 and 6). The abundance of the carbohydrate-binding module (CBM) was significantly decreased in treatments L-B and L-C on day 5, compared to the uninvaded control (*p* < 0.05, *t*-test; Fig. 6b). At the same time point, treatments E-A and M-A significantly decreased the abundance of the auxiliary activities family (AA) (*p* < 0.05, *t*-test; Fig. 6b). On day 60, the abundance of the polysaccharide lyases family (PL) increased in the treatment M-A but decreased in L-A and L-C (*p* < 0.05, *t*-test; Fig. 6b). Differences in the abundance of several classified genes related to compound degradation were investigated. The abundance of ligninolytic genes decreased in all treatments on day 5 except L-B, compared with the uninvaded control (*p* < 0.05, *t*-test; Fig. 6c). However, these differences were absent on day 60. The paired *t*-test between the log fold changes of functional capabilities (i.e., cellulolytic, hemicellulolytic, and ligninolytic) on days 5 and 60 indicates the recovery of these functions after 60 days (*p* < 0.05, paired *t*-test; Fig. 6d).

### Multiple traits of invasive communities predict invasion impacts

**Fig. 7:**
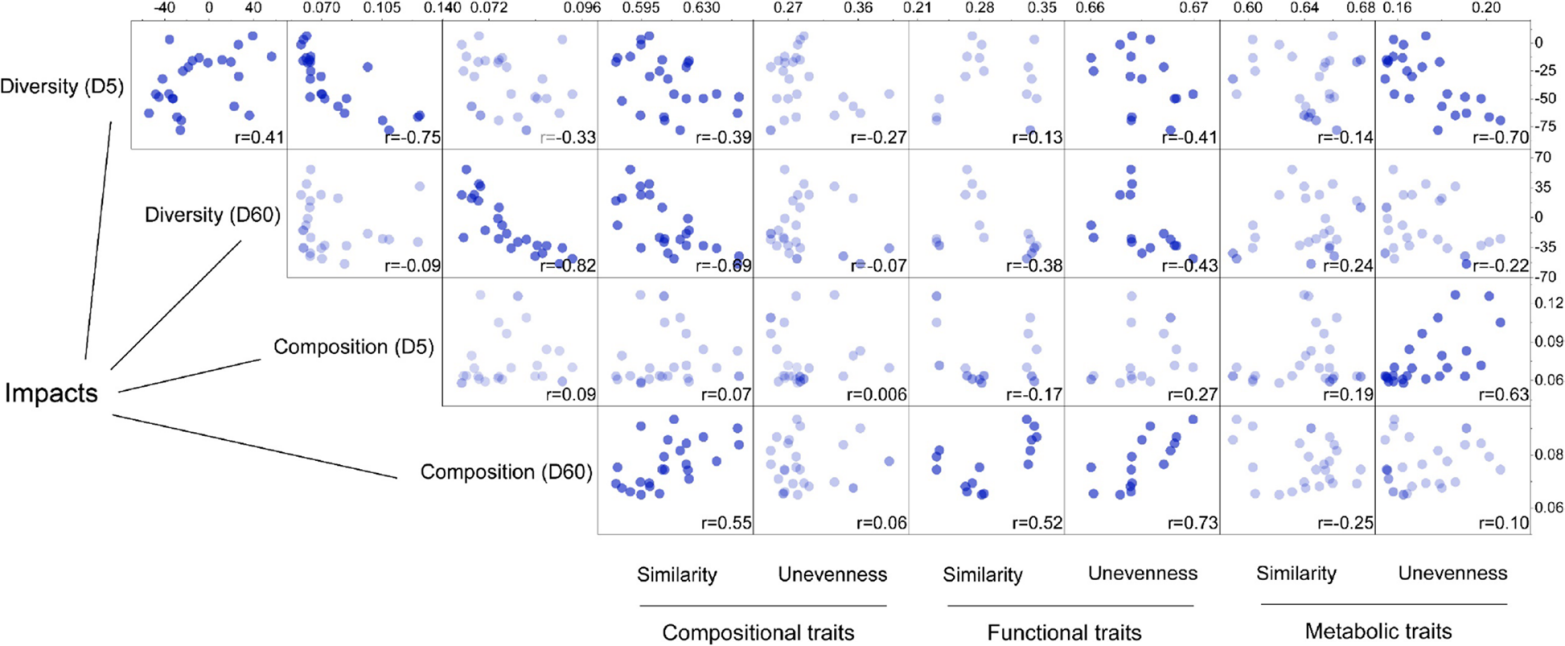
Multi-level traits of invasive communities predict invasion impacts. Pearson’s correlation matrix showing similarity and unevenness indexes of invasive communities predicting the invasion impact (change ratio (%) of richness and compositional dissimilarity in relation to uninvaded control) of coalescent communities at days 5 (D5) and 60 (D60). Points in darker colors indicate a significant correlation (*p* < 0.05). The compositional similarity (*n* = 27) was calculated based on the weighted UniFrac distance between invasive and resident communities at day 0. Functional (CAZy genes, *n* = 15) and metabolic (utilization of 71 C sources, *n* = 27) similarities were based on Bray-Curtis similarity between invasive and resident communities at day 0. Unevenness indexes of invasive communities were calculated as Strong’s dominance index. Treatment E-C was excluded as the outlier from this analysis because of the higher metabolic unevenness than other treatments

We correlated the compositional shift and change ratio (%) of richness (in relation to the uninvaded control) of coalescent communities on days 5 and 60 to multiple traits of invasive communities (Fig. 7). These traits were indexes of similarity and unevenness at the compositional (phylogenetic), functional (genes associated with carbohydrate metabolism), and metabolic (carbon metabolic profiling) levels, respectively. We found that the change ratio of the diversity of the coalescent community on day 5 was significantly and negatively correlated with compositional similarity, functional and metabolic unevenness of invasive communities. In the case of day 60, such correlations were observed for compositional similarity and functional unevenness of invasive communities. For the compositional shift on day 5, it was only significantly and positively correlated to the metabolic unevenness of invasive communities. However, the compositional shift on day 60 was significantly correlated both to the compositional (phylogenetic) similarity and functional similarity and unevenness indexes. In summary, we showed that at the beginning of coalescence, the impact on diversity and compositional shift is mainly due to metabolic unevenness, whereas at the end of the experiment, the impact on diversity and compositional changes could be explained mainly by compositional similarity and functional unevenness.

## Discussion

Microbial invasion driven by whole communities, known as community coalescence, occurs widely in natural ecosystems, including soils. Despite being an essential process in assembling soil microbial communities, our knowledge about the community-driven invasion in soils is limited. Here we used a multi-trait taxonomic and functional approach to understand the short and long-term consequences and potential mechanisms driving microbial community coalescence in the soil.

### The footprint of coalescences in soil microbial communities

As a biotic disturbance, community-driven invasions are expected to leave footprints in the native microbial communities and environment. Our results demonstrated that the coalescences significantly changed the successional trajectory of resident communities in relation to uninvaded control, and such coalescence-driven alterations were rooted in the changes in the diversity and composition across time. The richness of coalescent communities exhibited average declines from 16 to 65% on day 5 upon invasion. These declines imply the loss of resident taxa (below the detection limit). A similar phenomenon was also reported when a single invader (e.g., *E. coli* [23, 25]) and whole communities [28] were introduced into soil bacterial communities. A significant fluctuation in α diversity was observed among all treatments, including the uninvaded control. This might be partially due to the increased soil moisture when starting the experiment. In fact, we found that the incubation time, rather than invasion treatments, was more important in influencing the composition of soil microbial community. The probable reason is that we only introduced invasive taxa in a relatively small density (5% of resident taxa), and the influences of the subsequent biotic pressure were weaker than the change in soil moisture on the resident community. In this experiment, to allow the invading taxa to disperse relatively uniformly in the soil, the use of soil suspensions to generate coalescence is inevitable. In this way, invasive taxa will likely interact with native taxa more fully than directly mixing the soil [28]. Nonetheless, the significant change in community succession trajectories compared to the uninvaded control depended on the invasive community, and such change tended to be more pronounced after 60 days. Overall, this study shows that even when the size of the invasive community is small, and other factors can significantly affect the native community, the impact of community coalescence is not masked, highlighting the potential importance of community coalescence in soil ecosystems.

### The “lose-lose consequences” underlying community coalescences

The potential invasion-driven changes in coalescent communities were explored by classifying the survived/suppressed residents and invaders and investigating their contribution to the diversity and composition shifts observed for coalesced communities. In the present system, less than 1% of invaders survived (above the detection limit) post-invasions. The apparent low survival rate of the invasive taxa indicates that they encountered intense selective pressure after the coalescence. The abiotic pressure might be linked to resource availability in recipient soil, known for lacking labile carbon sources [33, 48]. Biotic factors could be directly associated with the competitive abilities of the communities. First, we found a significant positive correlation between the suppression rates of residents and invasive communities, which implies the direct and mutual antagonism between invaders and residents. Second, we found that suppressed resident subcommunities were phylogenetically closer to their invasive communities than those composed of survived taxa. A well-publicized and recently proven idea in microbial ecology is the competition-relatedness hypothesis, which suggests antagonism is primarily prevalent among phylogenetically and metabolically similar bacterial species [49, 50]. Thus, this provides the apparent clue that the suppression of resident taxa might be mainly due to the invader-imposed competition for metabolic resources.

Despite the low survival rate of invaders, our results illustrate that an average of 65% of resident taxa that survived in uninvaded control were suppressed to below the detection limit for all four dates. This result indicates that resident taxa can be suppressed despite the low survival rate of invaders, suggesting a “lose-lose” situation between invaders and resident species, meaning losses for both parties directly involved. Specifically, in a lose-lose scenario, even if some residents were suppressed, it would be challenging for the invaders to survive in large numbers. Moreover, we found that the suppression of resident taxa rather than invaders’ survival significantly correlated to invasion impacts. Therefore, the survival of invaders does not represent or predict the impact on the coalescent community, which needs to be paid attention to in follow-up studies, especially in a lose-lose scenario.

This study also demonstrates a cascading effect of invasion on the resident community, which goes beyond direct suppression of resident taxa. It has been widely accepted that competition between native species is also prevalent and helps sustain microbial coexistence [51–53]. As a result of community coalescence, the original microbial interactions within the resident community could be interrupted or demolished when resident taxa were partly suppressed. This leads to a further hypothesis that survived resident taxa might benefit from such an interruption due to the release of competitive pressure. Indeed, we find that the mean abundance (i.e., number of detected ASVs) of all survived residents was significantly higher in the coalescent community than in the original community or uninvaded control. These results suggest that (i) the suppression of some residents is beneficial to others, which may lead to a more pronounced community shift, and (ii) resident taxa rather than invaders would mainly occupy the remaining niche released by coalescences. Interestingly, a recent study found that some soil bacteria, such as *Bacillus* and *Burkholderia*, can significantly thrive when their competitors are depleted under removal treatments (e.g., antibiotics, filtration, and heat shock) [54]. These findings support the competition-driven niche segregation theory at the microbial community level. Competition between species results in diverging their niches in sympatry to reduce competition costs, a theory recently explored in the animal ecology [55]. More critically, our study highlights the pivotal role of community-driven invasion on soil microbial community assembly through changing microbial competition and coexistence.

### Predictability of coalescence impacts depends on both time and trait type

Resource competition is a common phenomenon among macro- and micro-organisms where two species occupying the same niche are expected to compete for identical resources [49, 56]. However, widespread functional redundancy leads to complex resource utilization spectra of the microbial community, forcing us to consider the traits of the entire community. Therefore, we introduced two indexes - trait similarity and unevenness - to represent microbial communities’ (potential) functional structure. Although multi-level traits can predict the coalescence impacts, we found that the different traits influenced the prediction at different time points. These results illustrate that resource competition is vital in coalescence, although the mechanism is complex. For example, metabolic unevenness is more crucial in promoting changes in the coalescent community on day 5. In contrast, the compositional similarity and functional unevenness mainly predict the invasion impact on day 60.

At the early coalescence stage, the invasion impact might be mainly caused by the depletion of several labile resources by specific invasive microorganisms. The Random Forest analysis further identified carbohydrates and carboxylic/acetic acid as core carbon sources as the most critical labile carbon sources in predicting the invasion impacts on day 5 (Supplementary Fig. S8). At this stage in the process of community coalescence, we expect that the direct influence (i.e., the interaction between invader and resident) posed by invaders might varied depending on invasive species, such as how quickly they compete for resources, the proportion of r-strategists in invasive communities, and the availability of niche for invaders. Indeed, a recent meta-analysis provides empirical evidence that increasing soil nutrient content can improve the effectiveness of microbial inoculants in mesocosm and field environments [57], which is aligned with microcosm results that resource addition promotes the invader’s survival [22].

Whereas the unevenness of specific metabolic traits largely explains the effects on community diversity and composition in response to early coalescence, they fail to explain the impact observed at late coalescence. At that stage, the unevenness of specific functional genes associated with enzymes involved in carbohydrate metabolism was highly correlated with compositional changes. These indicate metabolic traits could be more sensitive in predicting the coalescence impact at the early stage of coalescence while combining functional and compositional traits can improve the predictability for the long-term consequence. Overall, our study suggests that exploring community coalescence from a resource competition perspective is particularly important in agroecosystems for promoting colonization and managing the risk of microbial consortia inoculation.

We demonstrate the effect of community-driven invasions on microbial community functionalities. Based on shotgun metagenomic sequencing, we show that the ligninolytic capabilities of coalescent communities significantly decreased 5 days after the coalescence. This may be due to the suppression of native species. However, after 60 days, such differences in ligninolytic potential disappeared. Besides, the genes encoding for the degradation of plant-source compounds (lignin, hemicellulose, and cellulose) [58] respond differently to coalescences between days 5 and 60. These suggest the functional recovery and even preferences for compounds of plant origin in coalescent communities. Overall, we show that the functional capacities related to carbohydrate-active enzymes were mainly changed at the early stage of coalescence and showed no significant difference compared to the control at the late stage. This is inconsistent with the compositional traits and might be due to the high redundancy of carbohydrate-active enzyme genes in soil microbiomes.

## Conclusions

In conclusion, our study shows that microbial community-driven invasions can disturb the successional trajectory and functionalities of the soil native microbial community, largely depending on resource competition between invasive and resident taxa, even though in the lose-lose scenario (both invaders and residents have been suppressed). Residents surviving the coalescence can benefit from this competition and occupy niches released by the loss of some residents, which reshapes the coalescent community. In addition, we show that multi-level trait-based methods will help us study and predict the process and impact of community-driven invasions. This study represents an important step in investigating this common process in natural soil. Nonetheless, it is still challenging to distinguish other mechanisms underlying invasion impacts, such as microbial interactions through competition for nitrogen and phosphorus resources, antagonism through producing antibiotics, direct/indirect cooperation among species, and the effect of invaders’ necromass, as the importance of these mechanisms may vary in different coalescence scenarios. Additionally, whether and how quickly the invaded community can recover to being consistent with the non-invaded treatment remains to be studied. Furthermore, the evolutionary history and subsequent coevolution of members in the coalesced community may regulate the successional trajectory of the microbial community [27], promoting the understanding of microbial community succession in natural ecosystems and the application of beneficial microbial consortia in agricultural productions.

### Supplementary Information

**Additional file 1:** **Table S1.** The culturable bacterial density of the original resident and nine invasive communities. In the invasion experiment, the bacterial density of each soil suspension was adjusted to 5 % of that in the original soil. **Table S2.** Two-way ANOVA tests examining the effects of the factors time (dates), invasive community treatment, and their interaction on species richness. **Fig. S1.** The richness (a) and phylogenetic diversity (b) of the original resident community and nine invasive communities. Principal component analysis (PCoA) (c) and taxonomic profile (d) of the structure of the original resident community and nine invasive communities. The PCoA analysis is based on weighted Unifrac dissimilarity. Different letters above the bar indicate significant differences between treatments (*p* < 0.05, Tukey’s HSD). **Fig. S2.** ASV-set operation was used to distinguish survived/suppressed invaders and residents (a). Uppercase letters X, Y, and Z, represent constant resident subcommunity, invasive community, and the corresponding coalescent community. The numbers in each block are used to describe different groups of ASVs. Panel (b) shows the methods of set operation where the symbol “∩” and “–” means intersection and complement, respectively. As shown in the last panel (c), the suppression/survival rate was calculated, and the number of different taxa is indicated by the letter “n” followed by the corresponding numerical digits. **Fig. S3. **The successional path length of communities across 60 days. The path length (from day 0 to day 30 and day 30 to day 60) was calculated as weighted UniFrac distance (between two adjacent time points) and accumulated over time. Control means the uninvaded treatment. Different letters above boxes indicate significant differences between treatments (*p* < 0.05, Tukey’s HSD). The overall difference between coalescent treatments and control was estimated and shown as *p* value (Tukey’s HSD). **Fig. S4.** The survival of invaders after coalescences. (a) The survival rate of the invasive community under different treatments at four dates. The survival rate of the invasive community represents the percentage of invaders that survived in the soil after community coalescence. (b, c) Abundance (number of reads of each ASV) of survived invaders in invasive and coalescent communities. There were no significant differences in ASV abundance among the four dates (*p*> 0.05, one-way ANOVA). (d) The proportion of taxonomy of survived invaders. (e) The shared and unique survived invaders among four dates. D5, D15, D30, and D60 represent the days 5, 15, 30, and 60 after the coalescence, respectively. **Fig. S5.** Relationship between the ASV abundance of survived invaders in the invaded (coalescent) and invasive communities. The relationship was estimated using the Poisson Regression. a-d represent the days 5, 15, 30, and 60 after the coalescence, respectively. **Fig. S6.** Metabolic traits of invasive and resident communities. (a) Metabolic unevenness of original resident community (control) and nine invasive communities before the invasion. Different letters above the bar indicate significant differences between treatments (*p* < 0.05, Tukey’s HSD). Principal component analysis (PCoA) on the metabolic profiles of invasive and original resident communities (b) on day 0 (*p* < 0.001, Adonis) and coalescent communities and uninvaded control (c) on day 30 (*p* = 0.46, Adonis). **Fig. S7.** Principal component analysis (PCoA) on the CAZy genes profiles of invasive and original resident communities (a) on day 0 (*p* < 0.001, Adonis) and coalescent communities and uninvaded control (b) after coalescences (*p* > 0.05 for each date, Adonis). **Fig. S8.** Random Forest analysis showing core carbon sources causing community changes in richness and composition on day 5 (D5). Only carbon sources with a significant effect (*p*< 0.05, 27 carbon sources) were shown.

## Data Availability

All the raw sequencing data were deposited in the National Center for Biotechnology Information Sequence Read Archive under the accession number PRJNA843110.

## References

[1] Mallon CA, Elsas JDV, Salles JF (2015). Microbial invasions: the process, patterns, and mechanisms. Trends Microbiol.

[2] Roy HE, Bacher S, Essl F, Adriaens T, Aldridge DC, Bishop JDD (2019). Developing a list of invasive alien species likely to threaten biodiversity and ecosystems in the European Union. Glob Change Biol.

[3] Thakur MP, van der Putten WH, Cobben MMP, van Kleunen M, Geisen S (2019). Microbial invasions in terrestrial ecosystems. Nat Rev Microbiol.

[4] Nemergut DR, Schmidt SK, Fukami T, O’Neill SP, Bilinski TM, Stanish LF (2013). Patterns and processes of microbial community assembly. Microbiol Mol Biol Rev.

[5] Sorensen JW, Shade A (2020). Dormancy dynamics and dispersal contribute to soil microbiome resilience. Philos Trans R Soc Lond B Biol Sci..

[6] Rillig MC, Antonovics J, Caruso T, Lehmann A, Powell JR, Veresoglou SD (2015). Interchange of entire communities: microbial community coalescence. Trends Ecol Evol.

[7] Rillig MC, Lehmann A, Aguilar-Trigueros CA, Antonovics J, Caruso T, Hempel S (2016). Soil microbes and community coalescence. Pedobiologia.

[8] Röhl O, Graupner N, Peršoh D, Kemler M, Mittelbach M, Boenigk J (2018). Flooding duration affects the structure of terrestrial and aquatic microbial eukaryotic communities. Microb Ecol.

[9] Rocca JD, Simonin M, Bernhardt ES, Washburne AD, Wright JP (2020). Rare microbial taxa emerge when communities collide: freshwater and marine microbiome responses to experimental mixing. Ecology.

[10] Smith DJ, Timonen HJ, Jaffe DA, Griffin DW, Birmele MN, Perry KD (2013). Intercontinental dispersal of bacteria and archaea by transpacific winds. Appl Environ Microbiol.

[11] Litchman E (2010). Invisible invaders: non-pathogenic invasive microbes in aquatic and terrestrial ecosystems. Ecol Lett.

[12] Clark DR, Underwood GJC, McGenity TJ, Dumbrell AJ (2021). What drives study-dependent differences in distance–decay relationships of microbial communities?. Glob Ecol Biogeogr.

[13] Green J, Bohannan BJM (2006). Spatial scaling of microbial biodiversity. Trends Ecol Evol.

[14] Hanson CA, Fuhrman JA, Horner-Devine MC, Martiny JBH (2012). Beyond biogeographic patterns: processes shaping the microbial landscape. Nat Rev Microbiol.

[15] Olanrewaju OS, Babalola OO (2019). Bacterial consortium for improved maize (Zea mays L.) production. Microorganisms..

[16] Cortes-Tolalpa L, Norder J, van Elsas JD, Salles JF (2018). Halotolerant microbial consortia able to degrade highly recalcitrant plant biomass substrate. Appl Microbiol Biotechnol.

[17] Gravuer K, Scow KM (2021). Invader-resident relatedness and soil management history shape patterns of invasion of compost microbial populations into agricultural soils. Appl Soil Ecol..

[18] Liu X, Roux XL, Salles JF (2022). The legacy of microbial inoculants in agroecosystems and potential for tackling climate change challenges. iScience..

[19] Santos MS, Nogueira MA, Hungria M (2019). Microbial inoculants: reviewing the past, discussing the present and previewing an outstanding future for the use of beneficial bacteria in agriculture. Amb Express..

[20] Ahmad M, Pataczek L, Hilger TH, Zahir ZA, Hussain A, Rasche F (2018). Perspectives of microbial inoculation for sustainable development and environmental management. Front Microbiol..

[21] Mawarda PC, Le Roux X, van Dirk Elsas J, Salles JF (2020). Deliberate introduction of invisible invaders: a critical appraisal of the impact of microbial inoculants on soil microbial communities. Soil Biol Biochem..

[22] Mallon CA, Poly F, Le Roux X, Marring I, van Elsas JD, Salles JF (2015). Resource pulses can alleviate the biodiversity-invasion relationship in soil microbial communities. Ecology.

[23] Xing JJ, Sun SS, Wang HZ, Brookes PC, Xu JM (2020). Response of soil native microbial community to *Eschericia coli* O157:H7 invasion. Environ Pollut..

[24] Mallon CA, Le Roux X, van Doorn GS, Dini-Andreote F, Poly F, Salles JF (2018). The impact of failure: unsuccessful bacterial invasions steer the soil microbial community away from the invader’s niche. ISME J.

[25] Xing J, Jia X, Wang H, Ma B, Falcão Salles J, Xu J (2021). The legacy of bacterial invasions on soil native communities. Environ Microbiol.

[26] Amor DR, Ratzke C, Gore J (2020). Transient invaders can induce shifts between alternative stable states of microbial communities. Sci Adv.

[27] Castledine M, Sierocinski P, Padfield D, Buckling A (2020). Community coalescence: an eco-evolutionary perspective. Philos Trans R Soc Lond B Biol Sci.

[28] Huet S, Romdhane S, Breuil M-C, Bru D, Mounier A, Spor A (2023). Experimental community coalescence sheds light on microbial interactions in soil and restores impaired functions. Microbiome.

[29] Li SP, Tan JQ, Yang X, Ma C, Jiang L (2019). Niche and fitness differences determine invasion success and impact in laboratory bacterial communities. ISME J.

[30] van Elsas JD, Chiurazzi M, Mallon CA, Elhottovā D, Krištůfek V, Salles JF (2012). Microbial diversity determines the invasion of soil by a bacterial pathogen. Proc Natl Acad Sci U S A.

[31] Lechón-Alonso P, Clegg T, Cook J, Smith TP, Pawar S (2021). The role of competition versus cooperation in microbial community coalescence. PLoS Comput Biol.

[32] Jia X, Dini-Andreote F, Salles J (2020). Comparing the influence of assembly processes governing bacterial community succession based on DNA and RNA data. Microorganisms.

[33] Dini-Andreote F, Silva M, Triado-Margarit X, Casamayor EO, van Elsas JD, Salles JF (2014). Dynamics of bacterial community succession in a salt marsh chronosequence: evidences for temporal niche partitioning. ISME J.

[34] Mawarda PC, Lakke SL, van Elsas JD, Salles JF (2022). Temporal dynamics of the soil bacterial community following *Bacillus* invasion. iScience..

[35] Gohl DM, Vangay P, Garbe J, MacLean A, Hauge A, Becker A (2016). Systematic improvement of amplicon marker gene methods for increased accuracy in microbiome studies. Nat Biotechnol.

[36] Chen Y, Chen Y, Shi C, Huang Z, Zhang Y, Li S (2018). SOAPnuke: a MapReduce acceleration-supported software for integrated quality control and preprocessing of high-throughput sequencing data. Gigascience.

[37] Li D, Liu C-M, Luo R, Sadakane K, Lam T-W (2015). MEGAHIT: an ultra-fast single-node solution for large and complex metagenomics assembly via succinct de Bruijn graph. Bioinformatics.

[38] Hyatt D, Chen G-L, LoCascio PF, Land ML, Larimer FW, Hauser LJ (2010). Prodigal: prokaryotic gene recognition and translation initiation site identification. BMC Bioinformatics.

[39] Steinegger M, Söding J (2017). MMseqs2 enables sensitive protein sequence searching for the analysis of massive data sets. Nat Biotechnol.

[40] Cantarel BL, Coutinho PM, Rancurel C, Bernard T, Lombard V, Henrissat B (2009). The Carbohydrate-Active EnZymes database (CAZy): an expert resource for Glycogenomics. Nucleic Acids Res.

[41] Patro R, Duggal G, Love MI, Irizarry RA, Kingsford C (2017). Salmon provides fast and bias-aware quantification of transcript expression. Nat Methods.

[42] Mawarda PC, Mallon CA, Roux XL, van Elsas JD, Salles JF (2022). Interactions between bacterial inoculants and native soil bacterial community: the case of spore-forming *Bacillus* spp. FEMS Microbiol Ecol..

[43] Kassambara A. ggpubr: “ggplot2” Based Publication Ready Plots. 2020. Available from: https://CRAN.R-project.org/package=ggpubr.

[44] Oksanen J, Simpson GL, Blanchet FG, Kindt R, Legendre P, Minchin PR, et al. vegan: Community Ecology Package. 2022. Available from: https://CRAN.R-project.org/package=vegan.

[45] Bittinger K. abdiv: Alpha and Beta Diversity Measures. 2020. Available from: https://CRAN.R-project.org/package=abdiv.

[46] McMurdie PJ, Holmes S (2013). phyloseq: An R Package for Reproducible Interactive Analysis and Graphics of Microbiome Census Data. PLoS One.

[47] Archer E. rfPermute: Estimate Permutation p-Values for Random Forest Importance Metrics. 2022. Available from: https://CRAN.R-project.org/package=rfPermute.

[48] Dini-Andreote F, Stegen JC, van Elsas JD, Salles JF (2015). Disentangling mechanisms that mediate the balance between stochastic and deterministic processes in microbial succession. Proc Natl Acad Sci U S A.

[49] Russel J, Røder HL, Madsen J, Burmølle M, Sørensen S (2017). Antagonism correlates with metabolic similarity in diverse bacteria. Proc Natl Acad Sci U S A.

[50] Xia L, Miao Y, Cao A, Liu Y, Liu Z, Xinli S (2022). Biosynthetic gene cluster profiling predicts the positive association between antagonism and phylogeny in Bacillus. Nat Commun.

[51] Ghoul M, Mitri S (2016). The Ecology and Evolution of Microbial Competition. Trends Microbiol.

[52] Freilich S, Zarecki R, Eilam O, Segal ES, Henry CS, Kupiec M (2011). Competitive and cooperative metabolic interactions in bacterial communities. Nat Commun.

[53] Letten AD, Hall AR, Levine JM (2021). Using ecological coexistence theory to understand antibiotic resistance and microbial competition. Nat Ecol Evol.

[54] Romdhane S, Spor A, Aubert J, Bru D, Breuil M-C, Hallin S (2022). Unraveling negative biotic interactions determining soil microbial community assembly and functioning. ISME J.

[55] Reif J, Reifová R, Skoracka A, Kuczyński L (2018). Competition-driven niche segregation on a landscape scale: Evidence for escaping from syntopy towards allotopy in two coexisting sibling passerine species. J Anim Ecol.

[56] Craine JM, Dybzinski R (2013). Mechanisms of plant competition for nutrients, water and light. Funct Ecol.

[57] Liu X, Mei S, Salles JF (2023). Inoculated microbial consortia perform better than single strains in living soil: A meta-analysis. Appl Soil Ecol.

[58] López-Mondéjar R, Tláskal V, Větrovský T, Štursová M, Toscan R, da Nunes Rocha U (2020). Metagenomics and stable isotope probing reveal the complementary contribution of fungal and bacterial communities in the recycling of dead biomass in forest soil. Soil Biol Biochem..

