# Supplementary material for: Lose-lose consequences of bacterial community-driven invasions in soil
Source: Microbiome. 2024 Mar 18;12:57. doi: 10.1186/s40168-024-01763-7 (PMC10946201; doi:10.1186/s40168-024-01763-7)
Supplement: Supplementary file 2 — Additional file 1: Table S1. The culturable bacterial density of the original resident and nine invasive communities. In the invasion experiment, the bacterial density of each soil suspension was adjusted to 5 % of that in the original soil. Table S2. Two-way ANOVA tests examining the effects of the factors time (dates), invasive community treatment, and their interaction on species richness. Fig. S1. The richness (a) and phylogenetic diversity (b) of the original resident community and nine invasive communities. Principal component analysis (PCoA) (c) and taxonomic profile (d) of the structure of the original resident community and nine invasive communities. The PCoA analysis is based on weighted Unifrac dissimilarity. Different letters above the bar indicate significant differences between treatments (p < 0.05, Tukey’s HSD). Fig. S2. ASV-set operation was used to distinguish survived/suppressed invaders and residents (a). Uppercase letters X, Y, and Z, represent constant resident subcommunity, invasive community, and the corresponding coalescent community. The numbers in each block are used to describe different groups of ASVs. Panel (b) shows the methods of set operation where the symbol “∩” and “–” means intersection and complement, respectively. As shown in the last panel (c), the suppression/survival rate was calculated, and the number of different taxa is indicated by the letter “n” followed by the corresponding numerical digits. Fig. S3. The successional path length of communities across 60 days. The path length (from day 0 to day 30 and day 30 to day 60) was calculated as weighted UniFrac distance (between two adjacent time points) and accumulated over time. Control means the uninvaded treatment. Different letters above boxes indicate significant differences between treatments (p < 0.05, Tukey’s HSD). The overall difference between coalescent treatments and control was estimated and shown as p value (Tukey’s HSD). Fig. S4. The survival of invaders after coa [file 40168_2024_1763_MOESM1_ESM.docx]

**SUPPLEMENTARY INFORMATION**

Lose-lose consequences of bacterial community-driven invasions in soil

Xipeng Liu, Joana Falcão Salles ^*^

Microbial Ecology cluster, Genomics Research in Ecology and Evolution in Nature (GREEN), Groningen Institute for Evolutionary Life Sciences (GELIFES), University of Groningen, 9747 AG Groningen, The Netherlands

**^*^Corresponding authors**: Joana Falcão Salles ([j.falcao.salles@rug.nl](mailto:j.falcao.salles@rug.nl))

The supplementary information includes Supplementary Table S1-2 and Supplementary Fig. S1-8.

**Supplementary Tables**

**Table S1** The culturable bacterial density of the original resident and nine invasive communities. In the invasion experiment, the bacterial density of each soil suspension was adjusted to 5 % of that in the original soil.

| Soil | Culturable bacterial density (CFU g^-1^ soil) | Number of replicates |
| --- | --- | --- |
| Original | 7.0E+06 | 10 |
| E-A | 1.5E+09 | 3 |
| E-B | 2.4E+09 | 5 |
| E-C | 1.0E+09 | 4 |
| M-A | 2.4E+09 | 7 |
| M-B | 2.0E+09 | 3 |
| M-C | 1.1E+09 | 5 |
| L-A | 2.1E+09 | 5 |
| L-B | 7.4E+08 | 4 |
| L-C | 6.4E+08 | 3 |

**Table S2** Two-way ANOVA tests examining the effects of the factors time (dates), invasive community treatment, and their interaction on species richness.

| Source of variation | df | MS | *F* | *P* |
| --- | --- | --- | --- | --- |
| Richness |  |  |  |  |
| Time | 3 | 145994 | 3.88 | 0.013 |
| Invasive communities | 8 | 85281 | 2.27 | 0.033 |
| Time * Invasive communities | 24 | 146597 | 3.90 | < 0.001 |
| Error | 67 | 37614 |  |  |
|  |  |  |  |  |

**Supplementary Figures**


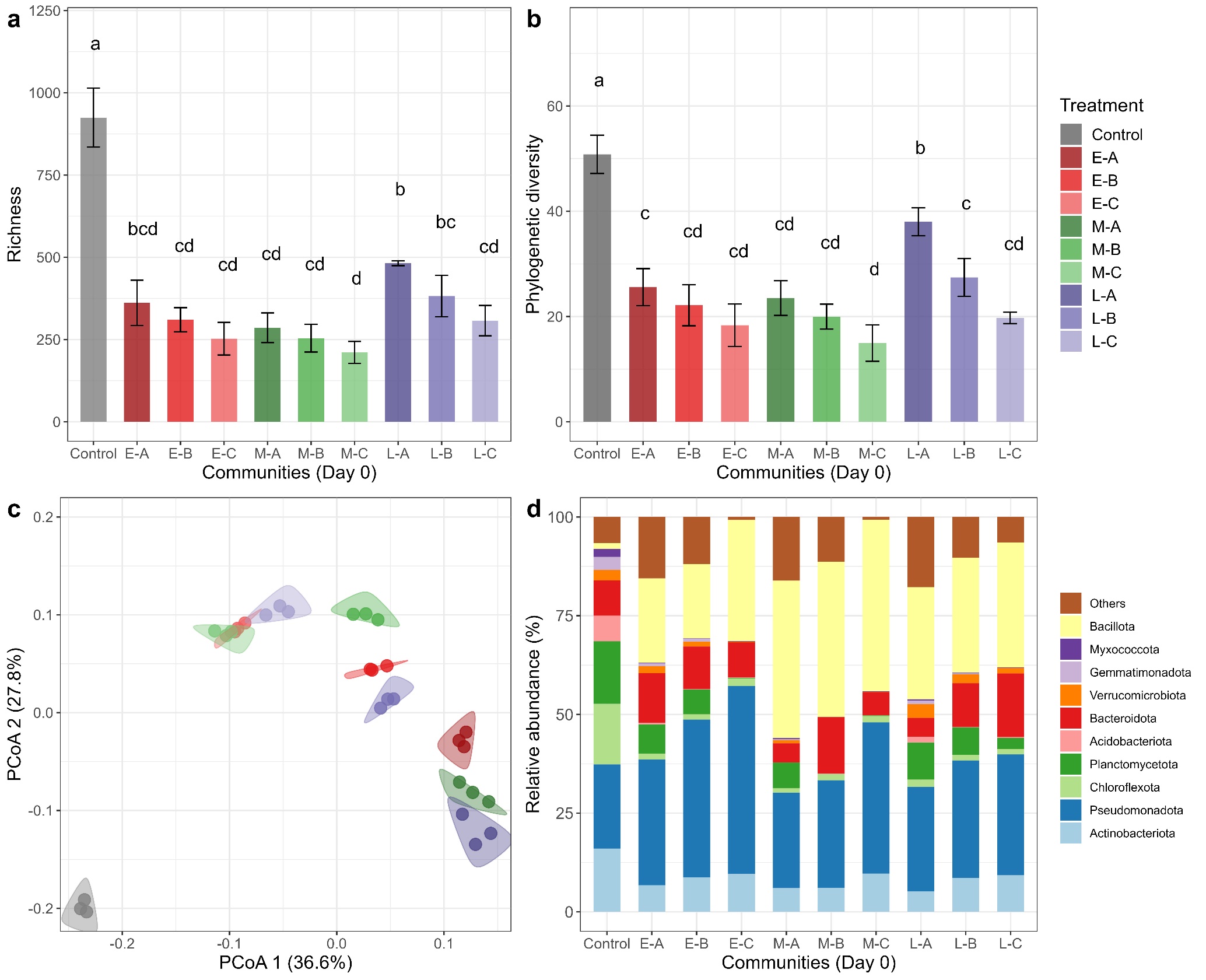


**Fig. S1** The richness (**a**) and phylogenetic diversity (**b**) of the original resident community and nine invasive communities. Principal component analysis (PCoA) (**c**) and taxonomic profile (**d**) of the structure of the original resident community and nine invasive communities. The PCoA analysis is based on weighted Unifrac dissimilarity. Different letters above the bar indicate significant differences between treatments (*p* < 0.05, Tukey’s HSD).


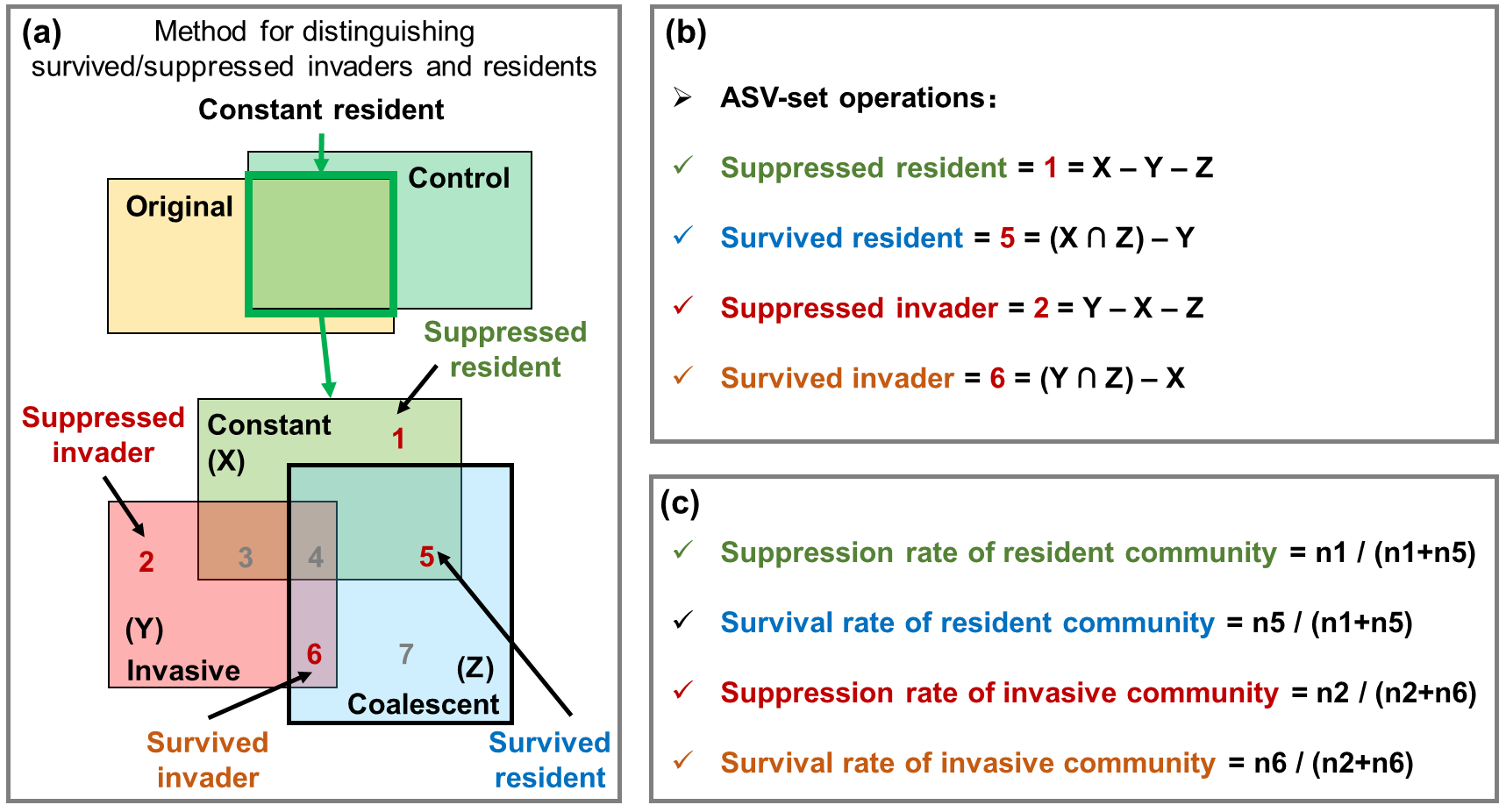


**Fig. S2** ASV-set operation was used to distinguish survived/suppressed invaders and residents (**a**). Uppercase letters X, Y, and Z, represent constant resident subcommunity, invasive community, and the corresponding coalescent community. The numbers in each block are used to describe different groups of ASVs. Panel (**b**) shows the methods of set operation where the symbol “∩” and “**–**” means intersection and complement, respectively. As shown in the last panel (**c**), the suppression/survival rate was calculated, and the number of different taxa is indicated by the letter “n” followed by the corresponding numerical digits.


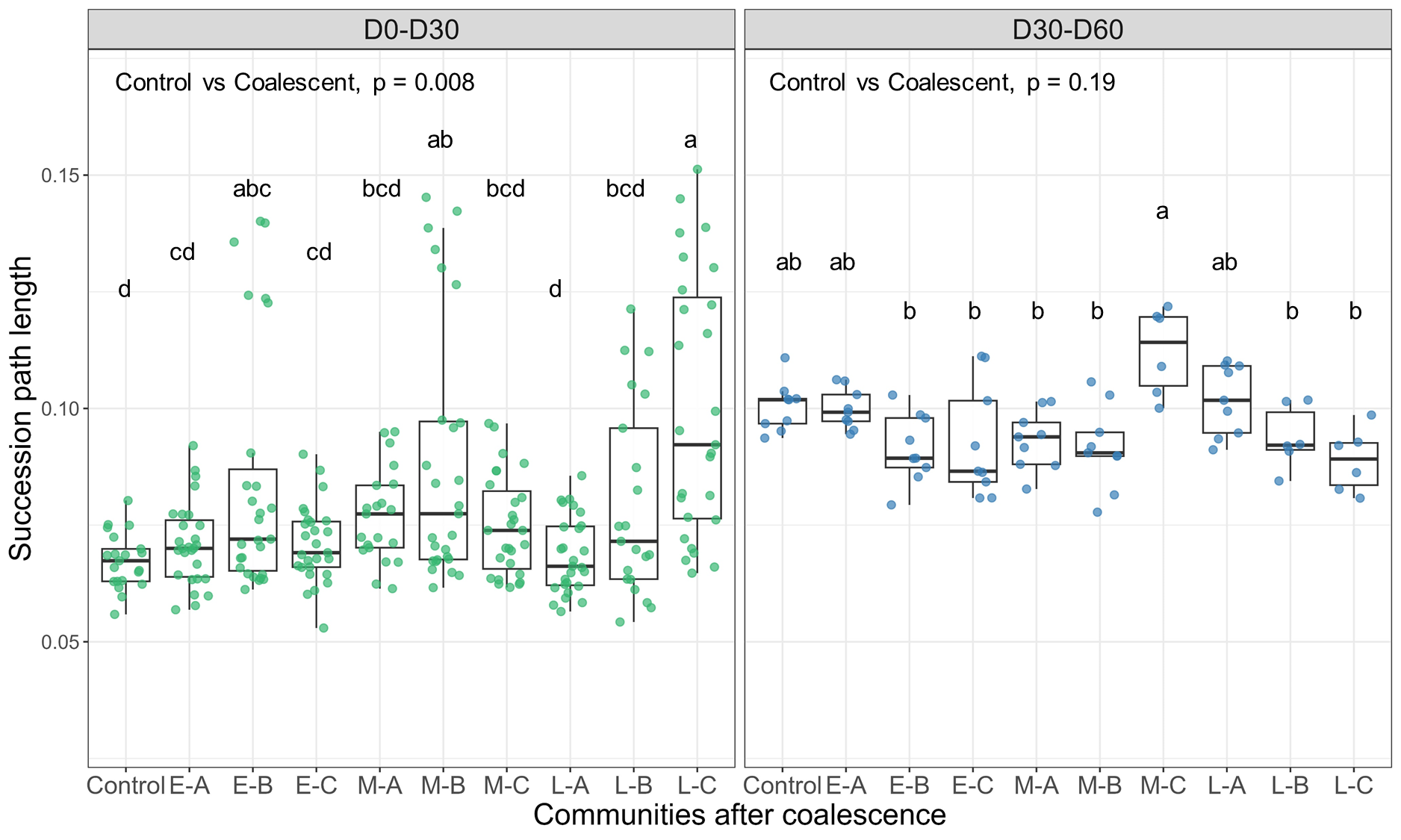


**Fig. S3** The successional path length of communities across 60 days. The path length (from Day 0 to Day 30 and Day 30 to Day 60) was calculated as weighted UniFrac distance (between two adjacent time points) and accumulated over time. Control means the uninvaded treatment. Different letters above boxes indicate significant differences between treatments (*p* < 0.05, Tukey’s HSD). The overall difference between coalescent treatments and control was estimated and shown as *p* value (Tukey’s HSD).


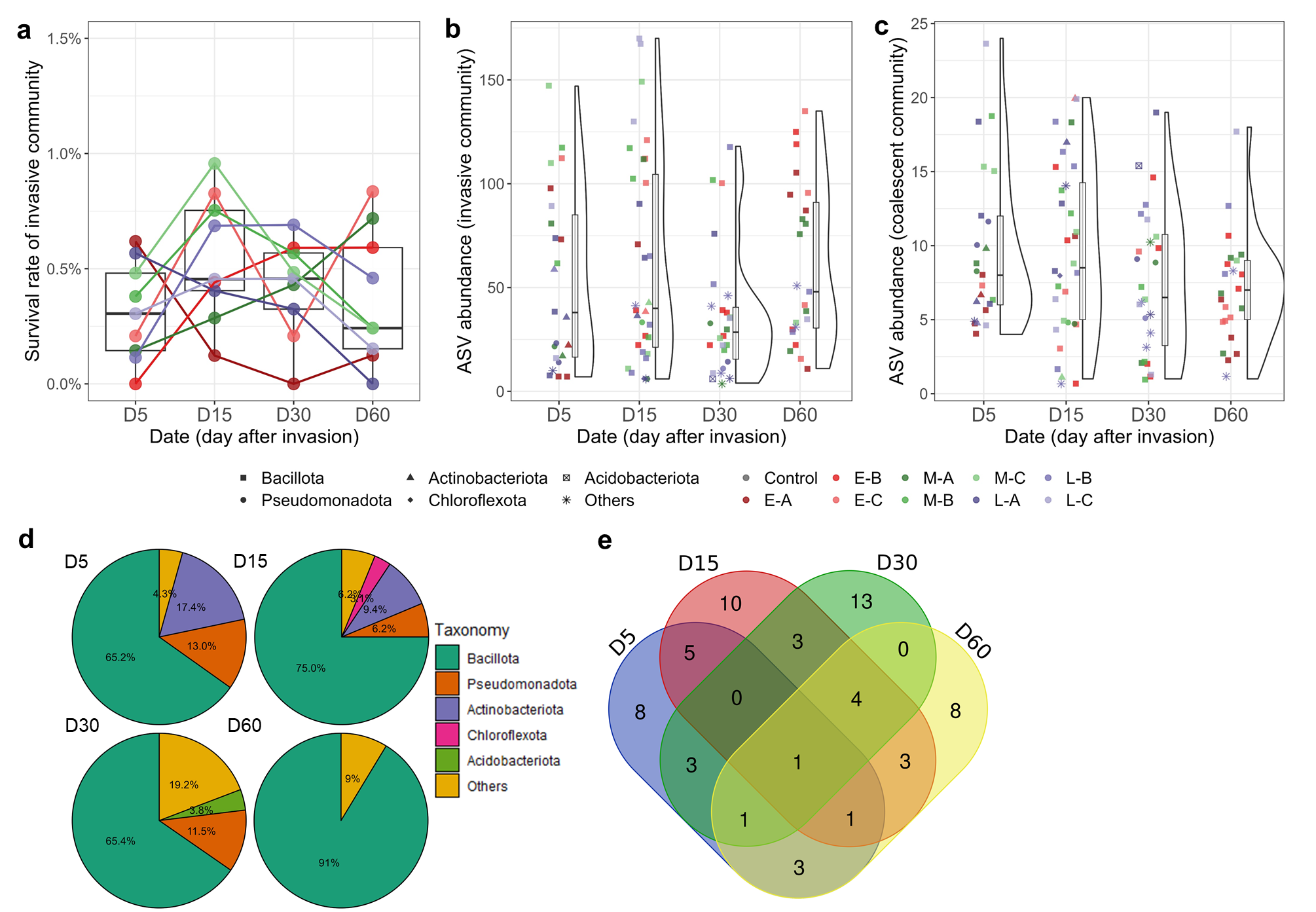


**Fig. S4** The survival of invaders after coalescences. (**a**) The survival rate of the invasive community under different treatments at four dates. The survival rate of the invasive community represents the percentage of invaders that survived in the soil after community coalescence. (**b, c**) Abundance (number of reads of each ASV) of survived invaders in invasive and coalescent communities. There were no significant differences in ASV abundance among the four dates (*p* > 0.05, one-way ANOVA). (**d**) The proportion of taxonomy of survived invaders. (**e**) The shared and unique survived invaders among four dates. D5, D15, D30, and D60 represent the Days 5, 15, 30, and 60 after the coalescence, respectively.


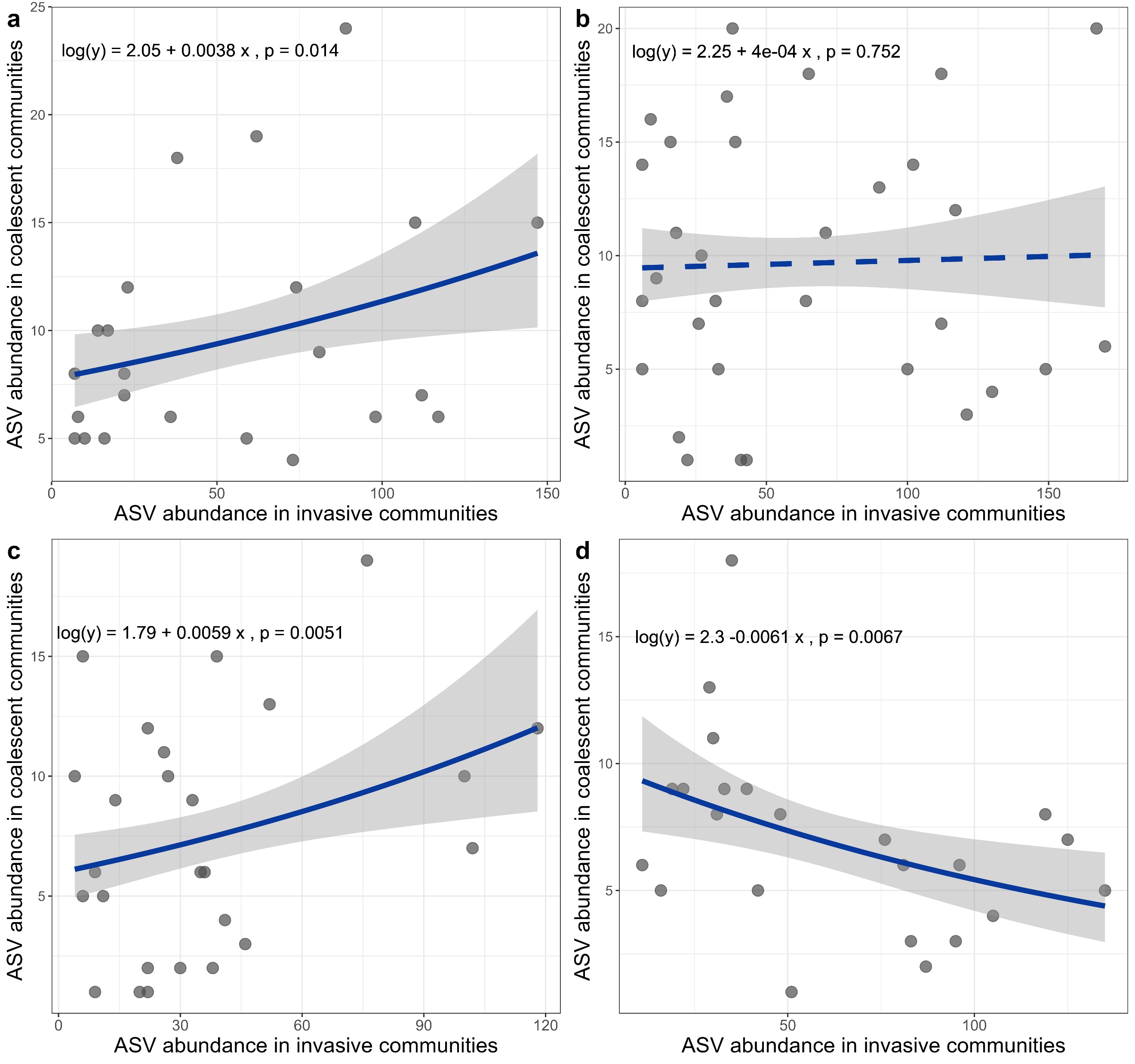


**Fig. S5** Relationship between the ASV abundance of survived invaders in the invaded (coalescent) and invasive communities. The relationship was estimated using the Poisson Regression. **a-d** represent the Days 5, 15, 30, and 60 after the coalescence, respectively.


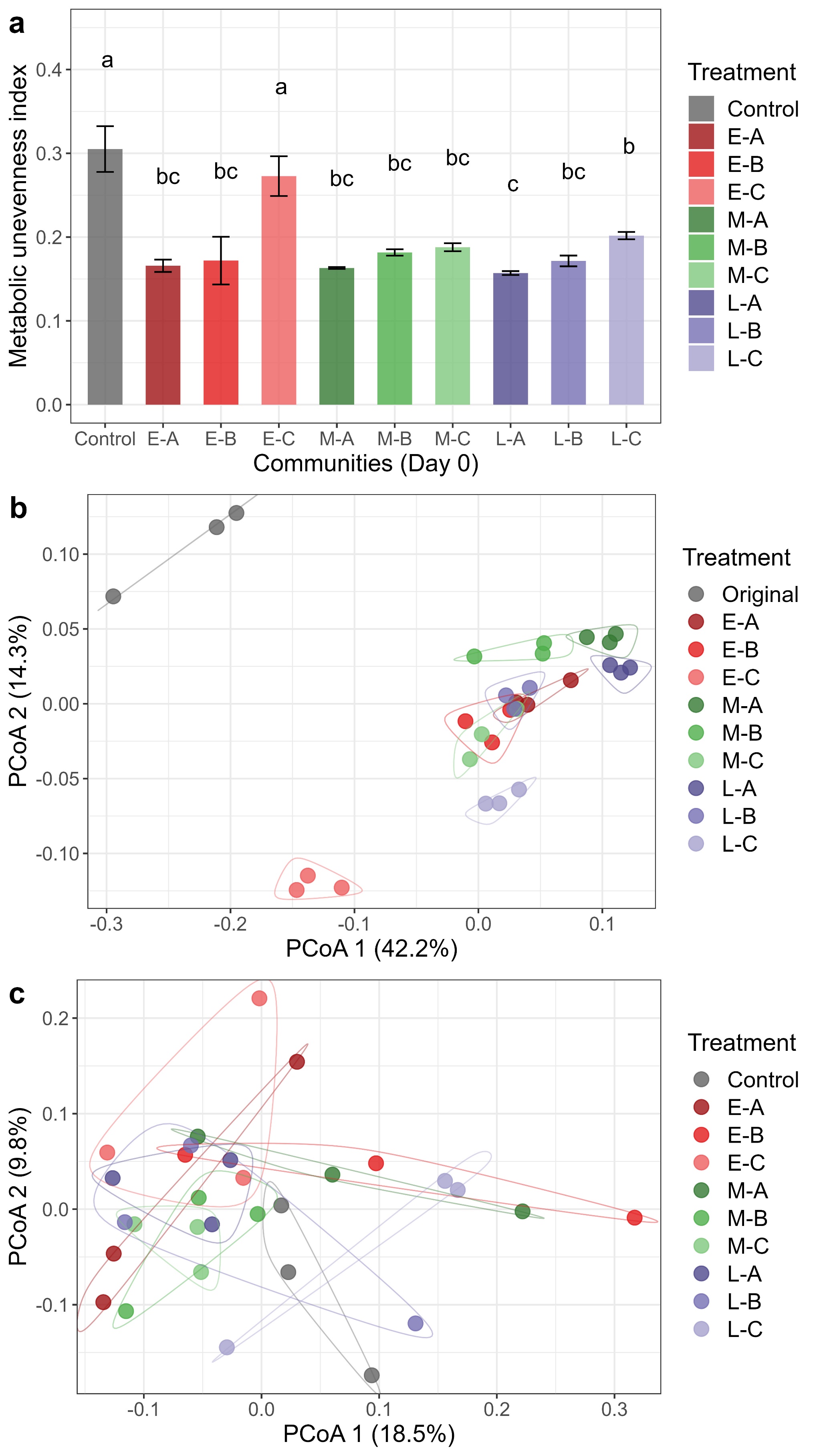


**Fig. S6** Metabolic traits of invasive and resident communities. (**a**) Metabolic unevenness of original resident community (control) and nine invasive communities before the invasion. Different letters above the bar indicate significant differences between treatments (*p* < 0.05, Tukey’s HSD). Principal component analysis (PCoA) on the metabolic profiles of invasive and original resident communities (**b**) on Day 0 (*p* < 0.001, Adonis) and coalescent communities and uninvaded control (**c**) on Day 30 (*p* = 0.46, Adonis).


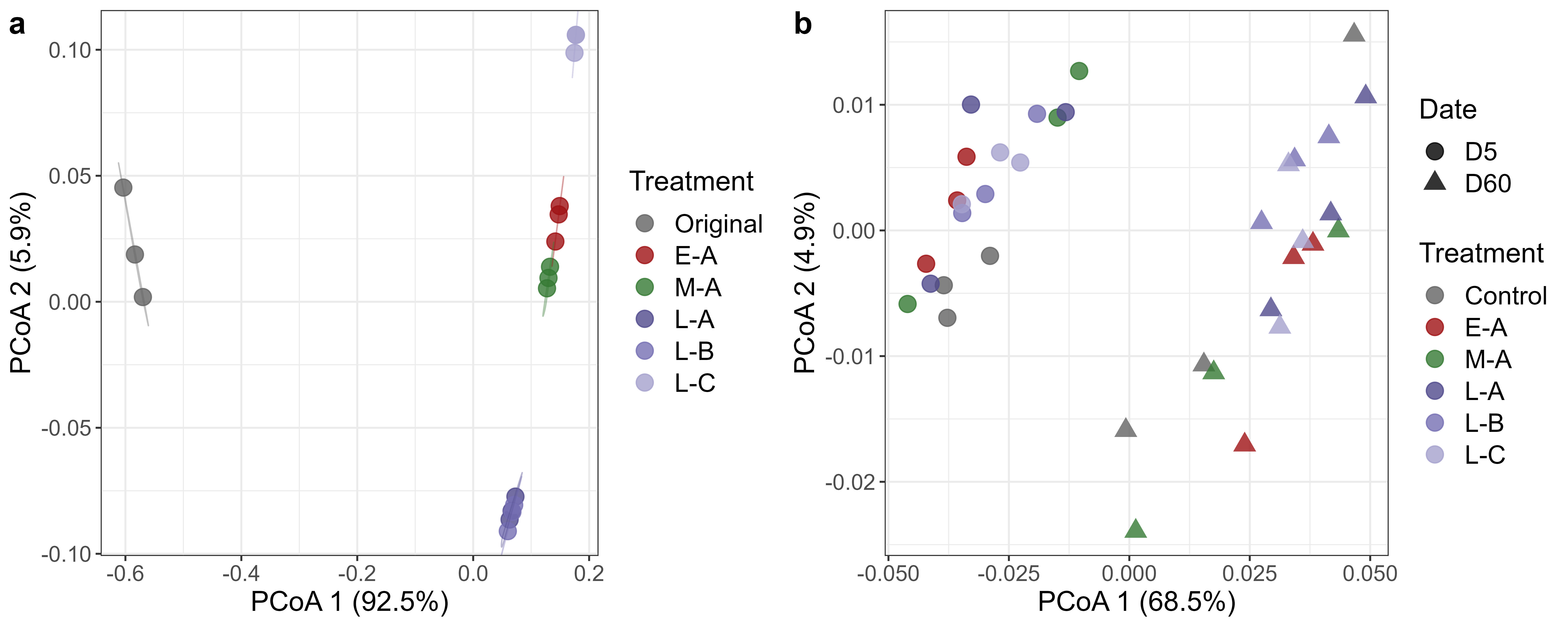


**Fig. S7** Principal component analysis (PCoA) on the CAZy genes profiles of invasive and original resident communities (**a**) on Day 0 (*p* < 0.001, Adonis) and coalescent communities and uninvaded control (**b**) after coalescences (*p* > 0.05 for each Date, Adonis).


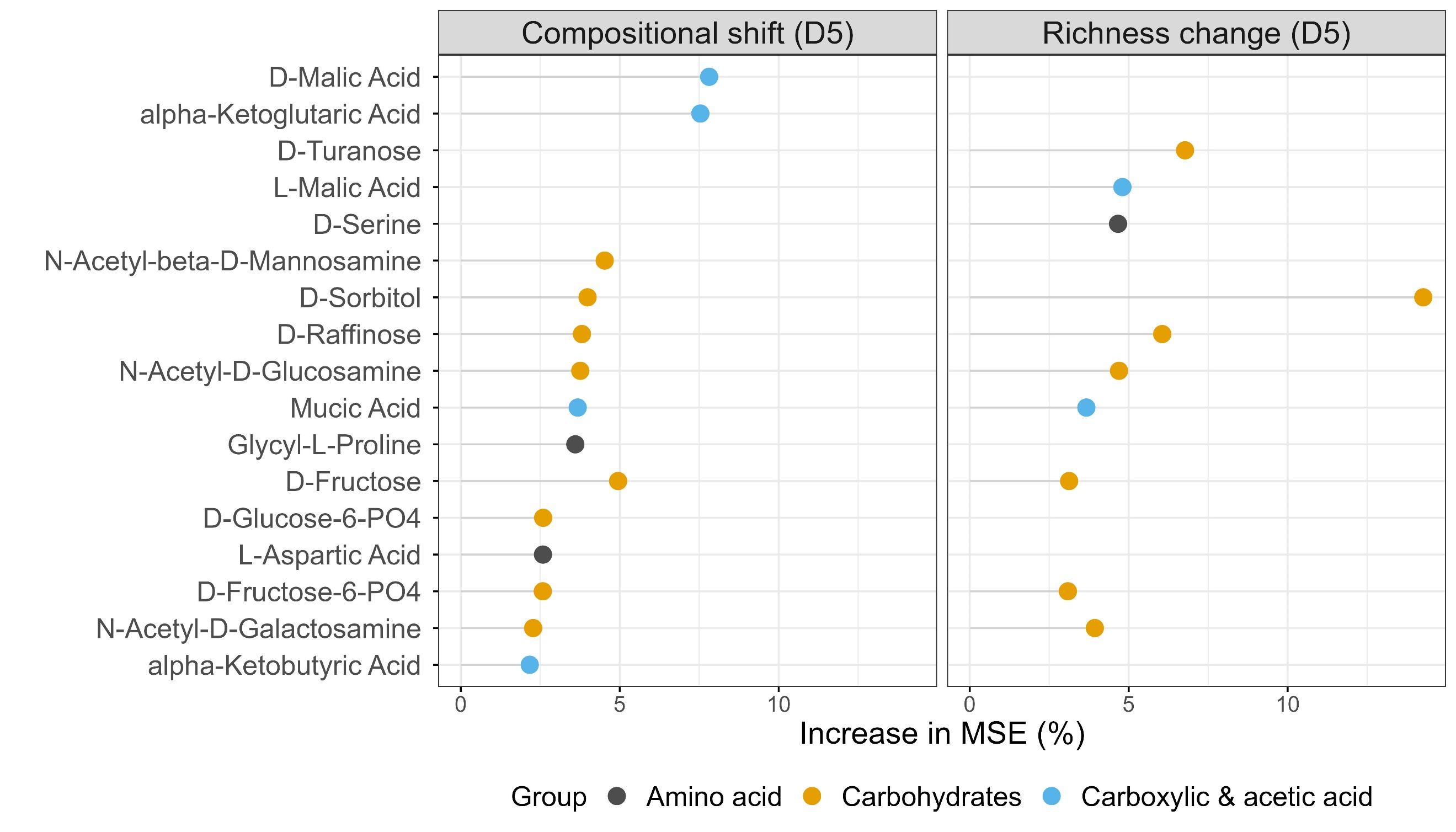


**Fig. S8** Random Forest analysis showing core carbon sources causing community changes in richness and composition on Day 5 (D5). Only carbon sources with a significant effect (*p* < 0.05, 27 carbon sources) were shown.
